# Molecular Mechanisms for the Adaptive Switching Between the OAS/RNase L and OASL/RIG-I Pathways in Birds and Mammals

**DOI:** 10.3389/fimmu.2018.01398

**Published:** 2018-06-20

**Authors:** Enguang Rong, Xiaoxue Wang, Hualan Chen, Chenghuai Yang, Jiaxiang Hu, Wenjie Liu, Zeng Wang, Xiaoyun Chen, Haixue Zheng, Juan Pu, Honglei Sun, Jacqueline Smith, David W. Burt, Jinhua Liu, Ning Li, Yinhua Huang

**Affiliations:** ^1^State Key Laboratory for Agrobiotechnology, China Agricultural University, Beijing, China; ^2^Animal Influenza Laboratory of the Ministry of Agriculture and National Key Laboratory of Veterinary Biotechnology, Harbin Veterinary Research Institute, Chinese Academy of Agricultural Sciences, Harbin, China; ^3^China Institute of Veterinary Drugs Control, Beijing, China; ^4^State Key Laboratory of Veterinary Etiological Biology and National Foot and Mouth Diseases Reference Laboratory, Lanzhou Veterinary Research Institute, Chinese Academy of Agricultural Sciences, Lanzhou, China; ^5^Key Laboratory of Animal Epidemiology and Zoonosis, Ministry of Agriculture, College of Veterinary Medicine, China Agricultural University, Beijing, China; ^6^The Roslin Institute and Royal (Dick) School of Veterinary Studies, University of Edinburgh, Edinburgh, United Kingdom

**Keywords:** birds, mammals, OASL, OAS/RNase L pathway, OASL/RIG-I pathway

## Abstract

Host cells develop the OAS/RNase L [2′–5′–oligoadenylate synthetase (OAS)/ribonuclease L] system to degrade cellular and viral RNA, and/or the OASL/RIG-I (2′–5′–OAS like/retinoic acid inducible protein I) system to enhance RIG-I-mediated IFN induction, thus providing the first line of defense against viral infection. The 2′–5′–OAS-like (OASL) protein may activate the OAS/RNase L system using its typical OAS-like domain (OLD) or mimic the K63-linked pUb to enhance antiviral activity of the OASL/RIG-I system using its two tandem ubiquitin-like domains (UBLs). We first describe that divergent avian (duck and ostrich) OASL inhibit the replication of a broad range of RNA viruses by activating and magnifying the OAS/RNase L pathway in a UBL-dependent manner. This is in sharp contrast to mammalian enzymatic OASL, which activates and magnifies the OAS/RNase L pathway in a UBL-independent manner, similar to 2′–5′–oligoadenylate synthetase 1 (OAS1). We further show that both avian and mammalian OASL can reversibly exchange to activate and magnify the OAS/RNase L and OASL/RIG-I system by introducing only three key residues, suggesting that ancient OASL possess 2–5A [p_x_5′A(2′p5′A)_n_; x = 1-3; n ≥ 2] activity and has functionally switched to the OASL/RIG-I pathway recently. Our findings indicate the molecular mechanisms involved in the switching of avian and mammalian OASL molecules to activate and enhance the OAS/RNase L and OASL/RIG-I pathways in response to infection by RNA viruses.

## Introduction

RNA viruses pose large challenges to human health and animal production with high mutation rates, rapid replication kinetics, and complex evolutionary dynamics (1, 2). To defend against virus infections, the host cellular innate immune system recognizes pathogen-associated molecular patterns with various pattern recognition receptors and activates a rapid antiviral response. After which, host cells secrete interferons (IFNs) to activate and stimulate a cascade of pathways for antiviral factors, including hundreds of IFN-stimulated genes (ISGs) (3, 4). Among ISGs, 2′–5′-oligoadenylate synthetase (OAS) plays a critical role in antiviral immunity by synthesizing 2–5As, which induces RNA degradation by activating a latent RNase (RNase L) pathway (5, 6).

The OAS repertoire is classed into four subfamilies that encode proteins of different isoforms in Metazoa. The small isoform [2′–5′-oligoadenylate synthetase 1 (OAS1)] consists of one copy of the enzymatic OAS domain, whereas the medium (OAS2) and large (OAS3) isoforms have one or two additional non-enzymatic OAS-like domains (OLDs) in the N-terminus. OASL presents an enzymatic (e.g., mouse Oasl2, mOasl2) or non-enzymatic OLD domain (e.g., human OASL, hOASL; mouse Oasl1, mOasl1) in the N-terminus and two tandem ubiquitin-like domains (UBLs) in the C-terminus (7–9). Recent evolutionary analyses suggested that adaptive selections to circumvent viral-encoded inhibitors in the OAS gene family have driven their functional diversity (10, 11). For example, all OAS subfamilies (OAS1-3) synthesize 2–5As to activate RNase L upon binding dsRNA (12, 13). However, *OAS1* prefers to bind cytosolic dsRNA with fewer than 20 bp and a 3′-single-stranded pyrimidine motif (5), whereas *OAS3* has a strong ability to bind long dsRNA (>50 bp) (12). Such adaptive change is even significant in the OASL subfamily, where the non-enzymatic hOASL mediates RIG-I activation to inhibit virus replication by mimicking polyubiquitin and upregulating the expression of IFNβ, TNFα, and IL-8, whereas the ortholog of hOASL in mouse (the non-enzymatic mOasl1) negatively regulates antiviral immunity by inhibiting the translation of IFN-regulatory factor 7 (IRF7) (14, 15). Moreover, the paralog of hOASL in mouse (mOasl2) synthesizes 2–5As activates RNase L and induces rRNA degradation after detecting dsRNA, similar to OAS1-3 (9).

Compared to mammals, birds have a contractive OAS family, which contains one member (OASL) in most birds belonging to the *Carinatae* group (e.g., ducks) and two members (OAS1 and OASL) in a few birds belonging to the *Ratitae* group (e.g., ostriches). Sequence alignment of 22 avian OASL molecules showed that they hold three conserved aspartic acid (D, homologous to D75-D77-D148 in human OAS1) residues, which serve as metal ion ligands and are required to activate the enzymatic activity to synthesize 2–5As (16). However, whether and how birds (especially *Carinataes*) can recognize divergent RNA viruses to activate the OAS/RNase L system and/or enhance the OASL/RIG-I system using one OAS member (OASL) (where mammals do it with a functionally diverse OAS family), is largely unknown.

**Figure 1:**
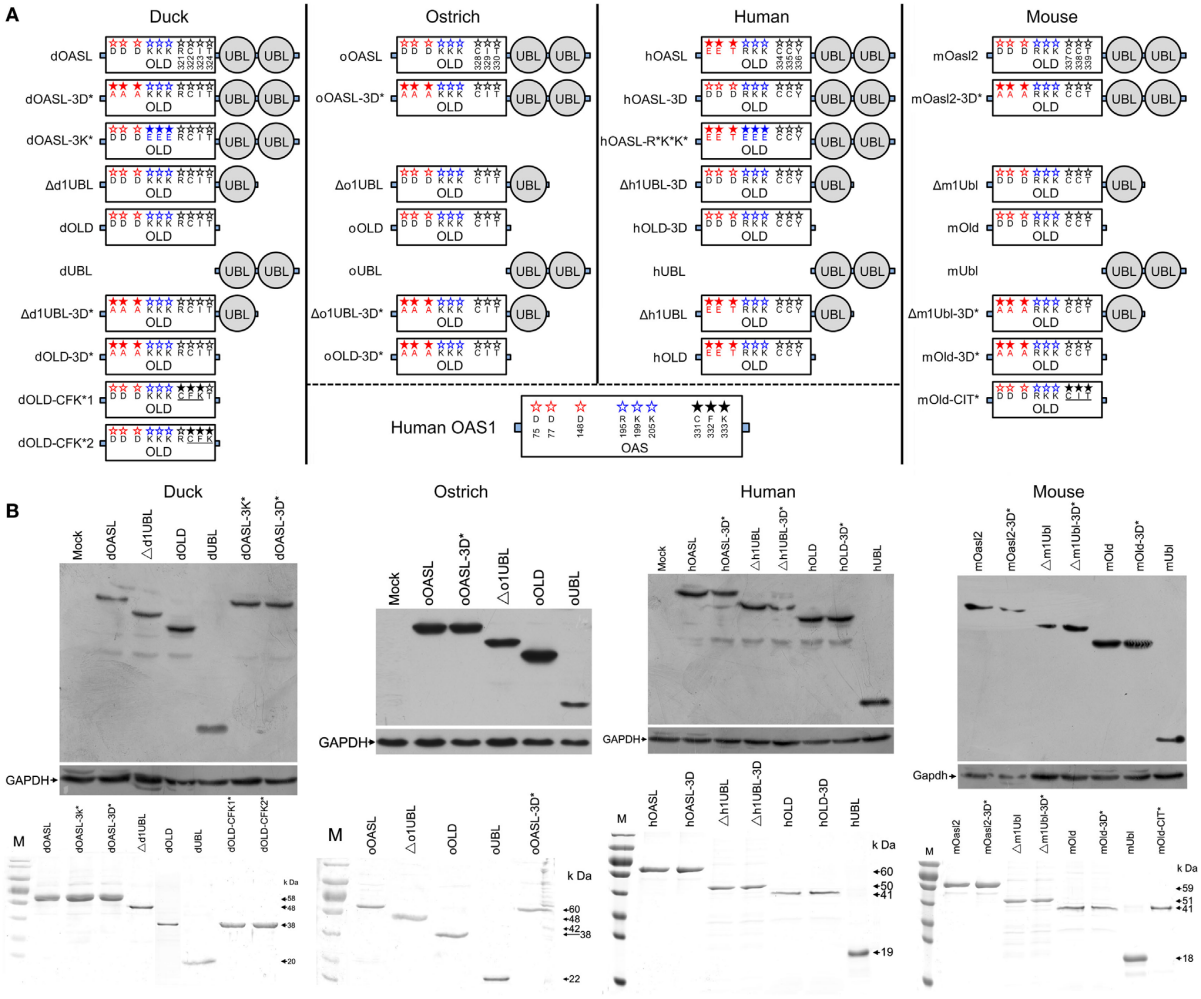
Domain organizations, purification, and expression in cells of duck, ostrich, human, and mouse OASL proteins and their mutants. **(A)** Schematic diagram of OASLs, their mutants and truncations. Red, green, and black letter in OLD domain mean amino acids being homologous to D75-D77-D148, R195-K199-K205, and C331-F332-K333 (CFK motif) of human 2′–5′-oligoadenylate synthetase 1, respectively. **(B)** Western blot analyses of OASLs, their mutants and truncations in DF1 (duck and ostrich) or A549 (human and mouse) cells (up), and His-tagged recombinant proteins (down). Western blot analysis was detected using Flag or c-Myc antibody. GAPDH (1:5,000) was used as a protein loading control. The purified proteins were analyzed by SDS-PAGE.

Here, we find that avian OASLs activate and enhance the OAS/RNase L pathway to inhibit replication of a positive single-stranded RNA virus, two strains of double-stranded RNA viruses, and four strains of negative single-stranded RNA viruses, which requires both their OLD and UBL domains. This differs from the situation in mammals, where one mammalian OASL (mOasl2) and one mammalian OASL mutant (hOASL-3D) activate and magnify the OAS/RNase L pathway to inhibit viral replication with their OLD domains like *OAS1* (Figure 1). Upon introduction of mutations at three D residues homologous to D75-D77-D148 of hOAS1, avian OASL-3D*, and mOasl2-3D* lose the ability to synthesize 2–5As, enhance the RIG-I antiviral activation, and upregulate the expression of many genes downstream of RIG-I in a virus- and UBL-dependent manner, similarly to hOASL (Figure 1). These results indicated that avian and mammalian OASLs could be an effective target for alternative regulation of the OAS/RNase L and OASL/RIG-I pathways during viral infection.

## Results

### Duck and Ostrich OASL Proteins Lead to Resistance to Infection by a Broad Range of RNA Viruses

**Figure 2:**
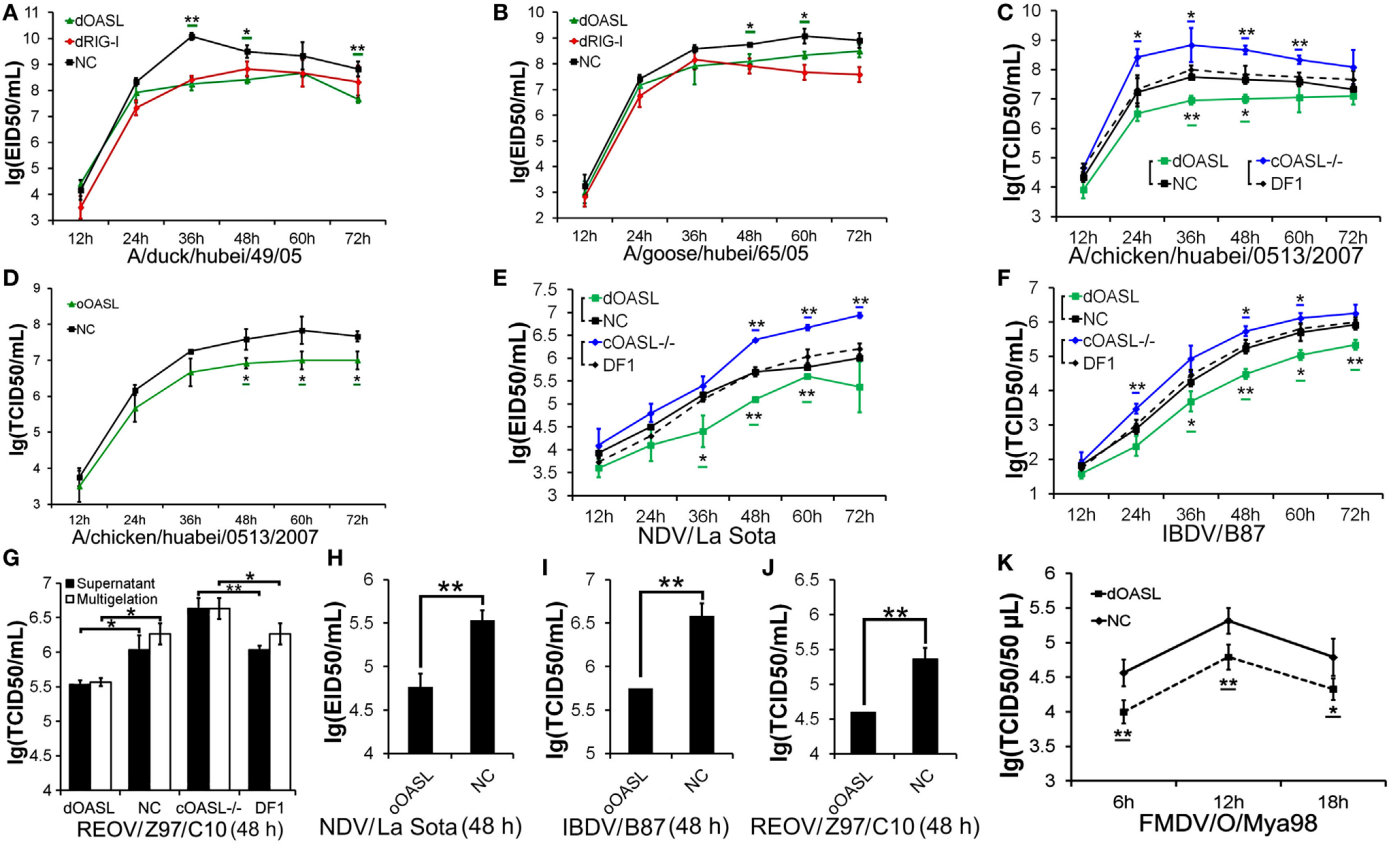
Duck and ostrich OASL proteins inhibit a broad range of RNA virus replications and the loss of chicken OASL enhances these RNA virus replications. Cells infected with virus were harvested at the indicated time points and subjected to EID50 assays or TCID50 assays on MDCK, DF1, Vero, or BHK21 cells (two–tailed Student’s *t*-test, *n* = 3). The data are expressed as the mean ± SD. *P < 0.05; **P < 0.01. **(A,B)** DF1^dOASL+/+^ cells have fewer DK/49 **(A)** and GS/65 **(B)** virus. DF1^dRIG-I+/+^ cells were a positive control for antiviral activity. **(C)** DF1^dOASL+/+^ cells exhibited lower level than NC cells, while DF1^OASL−/−^ (chicken OASL-deficient DF1) cells had higher level than wild-type DF1 cells, of CK/0513 virus. NC is chicken DF1 cell expressing empty vector. **(D)** DF1 cells expressing oOASL exhibited lower level of CK/0513 virus titers. **(E–G)** DF1^dOASL+/+^ cells produced fewer, while DF1^OASL−/−^ cells had more replication of NDV **(E)**, IBDV **(F)**, and REOV **(G)** virus. **(H–J)** DF1 cells expressing oOASL showed a severe reduction in virus titers of NDV **(H)**, IBDV **(I)**, and REOV **(J)** virus. **(K)** IBRS2 cells expressing dOASL had significantly lower FMDV virus titer.

Duck *RIG-I* can detect influenza A virus and induces an antiviral response in chicken embryonic fibroblasts (DF1) cells, where the RIG-I is absent (17). To determine the role of avian OASL in the immune response, we compared viral replication of a highly pathogenic (A/duck/Hubei/49/05, DK/49) and a weakly pathogenic (A/goose/Hubei/65/05, GS/65) H5N1 virus in DF1 cells expressing duck *OASL* (DF1^dOASL+/+^) to the corresponding in duck *RIG-I* recovery-expression DF1 cells (DF1^dRIG-I+/+^). Interestingly, DF1^dOASL+/+^ and DF1^dRIG-I+/+^ cells showed comparatively lower levels of DK/49 and GS/65 virus titers compared to DF1 cells expressing empty vectors, supporting that duck *OASL* can efficiently prevent viral infection, similarly to duck *RIG-I* (Figures 2A,B). We further found that DF1^dOASL+/+^ cells have a reduced expression of the matrix gene vRNA and mRNA of the DK/49 virus using a strand-specific real-time RT-PCR methods (18), implying that the dOASL may affect virus transport and transcription (Figure S1 in Supplementary Material). We then generated OASL-deficient DF1 cells (DF1^OASL−/−^) using the CRISPR/Cas9-mediated genome editing method (Figure S2 in Supplementary Material) (19). As expected, DF1^dOASL+/+^ cells had a significantly lower level of the CK/0513 (A chicken/huabei/0513/2007) H5N1 virus, whereas DF1^OASL−/−^ cells showed a significantly higher level of the CK/0513 virus compared to DF1 cells expressing an empty vector (Figure 2C). Similarly, we found that ostrich OASL (oOASL), representing ancient avian OASLs (20), significantly inhibited the replication of CK/0513 virus in DF1 cells (Figure 2D; Figure S1 in Supplementary Material), supporting that avian OASLs play a critical role in the immune response to influenza A viruses, similarly to some mammalian OASLs.

We next investigated the antiviral activity of avian OASLs against diverse viruses. Interestingly, the expression of either dOASL or oOASL in DF1 cells significantly reduced the replication of another negative single-stranded RNA virus (Newcastle disease virus, NDV/La Sota) and two double-stranded RNA viruses (infectious bursal disease virus, IBDV/B87 and respiratory enteric orphan virus, REOV/Z97/C10). In contrast, the absence of OASL in DF1 cells significantly enhanced the viral replication of the above three viruses (Figures 2E–J). Further analysis indicated that dOASL inhibited the replication of a positive single-stranded RNA virus (Foot-and-mouth disease virus, FMDV/O/Mya) in porcine kidney cells (IBRS2) (Figure 2K). However, neither dOASL nor oOASL reduced replication of two strains of double-stranded DNA virus in DF1 cells (fowlpox virus, FPV/CVCC/AV1003) or in porcine kidney epithelial (PK15) cells (pseudorabies virus, PRV/Henan/2014) (Figure S3 in Supplementary Material). Taken together, avian OASL shows antiviral activity against a wide range of RNA viruses but not against DNA viruses.

### Duck and Ostrich OASL Proteins Activate and Magnify the OAS/RNase L Pathway to Induce Viral RNA Degradation Similarly to Mouse Oasl2

**Figure 3:**
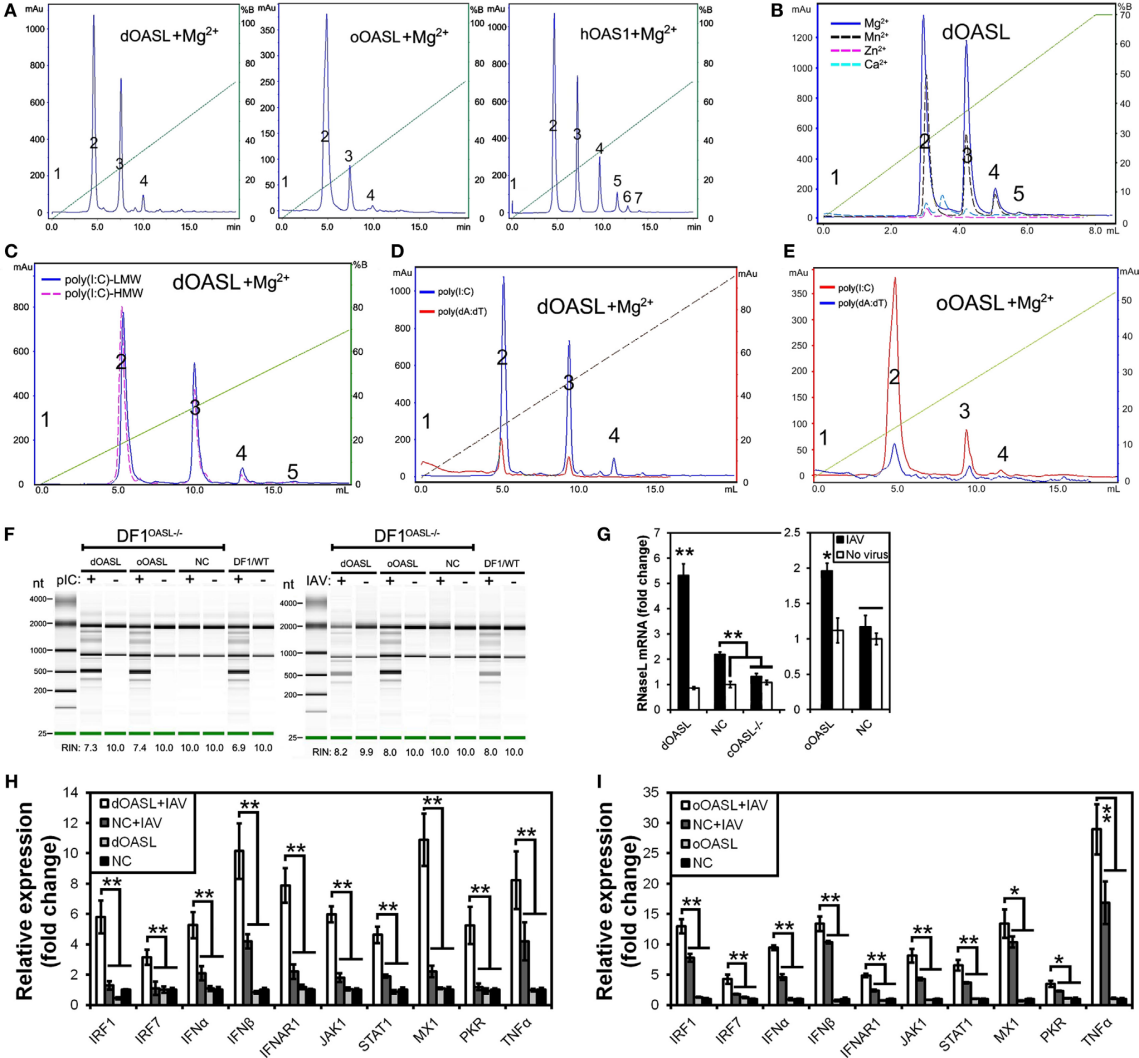
Duck and ostrich OASL proteins synthesize 2–5A to activate and enhance the OAS/RNase L pathway degrading cellular and viral RNA. The 2–5A synthetase reaction was treated with alkaline phosphatase and separated using a Mono Q column. hOAS1 was a positive control for 2–5A activity. Numbers in the elution profiles of A–E represent adenylate, pppApA, pppA(pA)_2_, pppA(pA)_3_, pppA(pA)_4_, pppA(pA)_5_, pppA(pA)_6_, respectively. NC is chicken OASL-deficient DF1 cell expressing empty vector. WT is parent DF1 cell. “RIN” is the RNA integrity number. Gene expressions were calculated relative mRNA level to that of *GAPDH* and presented as fold change against the corresponding of NC without CK/0513 infection (two-tailed Student’s *t*-test, n = 3). The data are expressed as the mean ± SD. *P < 0.05; **P < 0.01. **(A–E)** 2–5As produced by dOASL or oOASL with pIC **(A)**, dOASL with different divalent cations **(B)**, dOASL with HMW or LMW **(C)**, dOASL with pAT **(D)**, oOASL with pAT **(E)**. **(F)** rRNA cleavage induced by dOASL or oOASL in DF1^OASL−/−^ cells transfected with pIC (5 µg/mL) for 4 h or infected with CK/0513 (multiplicity of infection = 1) for 18 h. **(G–I)** dOASL and oOASL significantly increased the expression of *RNase L* **(G)** and 10 genes **(H,I)** related to IFN signaling after infection with CK/0513 virus in DF1^OASL−/−^ cells, respectively.

To investigate whether avian OASL activates the OAS/RNase L pathway to decay viral RNA, we first examined the 2–5A synthesis activities of duck (58 kDa) and ostrich (60 kDa) recombinant OASL proteins through a heat-inactivated 2–5A synthetase reaction. As expected, both dOASL and oOASL produced superimposed elution profiles with more than three peaks using poly(I:C) (pIC) as an activator, supporting that avian OASL synthesizes dimeric (pppApA), trimeric (pppApApA), and longer oligomers like human OAS1 (hOAS1) and mOasl2 (Figure 3A). Among four types of tested divalent cations, Mg^2+^ and Mn^2+^ stimulated the 2–5A activity of dOASL at a high level, whereas Zn^2+^ and Ca^2+^ stimulated this activity only at a low level (Figure 3B). Furthermore, both low (an average size of 0.2–1 kb) and high (an average size of 1.5–8 kb) weight pIC stimulated the 2–5A activity of dOASL and oOASL at a high level (Figure 3C), while poly(dA:dT) (pAT, a surrogate for dsDNA viruses) stimulated the 2–5A activity of dOASL and oOASL at a low level (Figures 3D,E).

**Figure 4:**
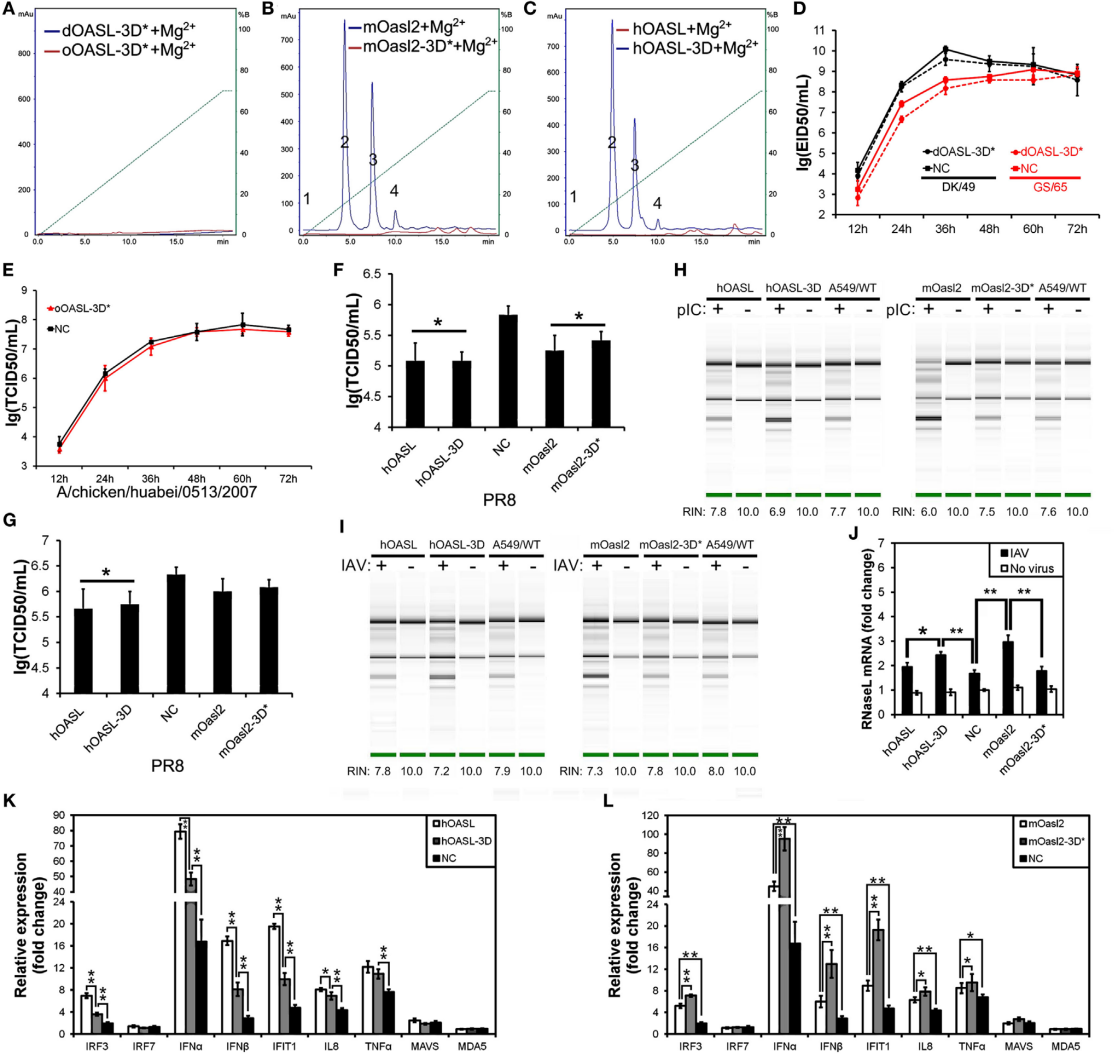
Three conserved sites of duck, ostrich, human, and mouse OASL being homologous to D75-D77-D148 of human 2′–5′-oligoadenylate synthetase 1 are a button for switching on/off the OAS/RNase L pathway. The 2–5A synthetase reaction was treated with alkaline phosphatase and separated using a Mono Q column. Gene expression in cells were calculated relative mRNA level to that of *GAPDH* and presented as fold change against the corresponding of A549 cells expressing empty vector (NC) without PR8 virus infection (two-tailed Student’s *t* test, *n* = 3). The data are expressed as the mean ± SD. *P < 0.05; **P < 0.01. **(A–C)** The elution profiles produced by dOASL-3D* or oOASL-3D* **(A)**, mOasl2 or mOasl2-3D* **(B)**, and hOASL-3D or hOASL proteins **(C)**. **(D,E)** dOASL-3D* and oOASL-3D* did not inhibit the DK/59 and GS/65 in DF1 cells **(D)** or CK/0513 **(E)** virus replication in DF1^OASL−/−^ cells, whose *RIG-I* is naturally absent. **(F,G)** hOASL-3D and mOasl2 slightly or significantly inhibited the replication of PR8 [multiplicity of infection (MOI) = 0.001] virus in A549 **(F)** or HeLa **(G)** cells. **(H,I)** rRNA cleavage induced by hOASL-3D and mOasl2 in A549 cells stimulated with pIC **(H)** (500 ng/mL) or infected with PR8 virus **(I)** MOI = 1. **(J–L)** hOASL-3D and mOasl2 significantly increased the expression of *RNase L* **(J)** and six genes **(K,L)** related to IFN signaling upon infection with PR8 virus (MOI = 0.001).

To test whether avian OASL activates RNase L to degrade RNA with their 2–5A products, we examined the rRNA integrity in different DF1 cells using a rRNA cleavage assay after induction with pIC. Expectedly, parental DF1 cells and both dOASL or oOASL recovery-expression DF1^OASL−/−^cells (transfected with dOASL or oOASL) had a low rRNA integrity number (RIN 6.9–7.4), whereas DF1^OASL−/−^ cells had a high RIN (10.0) (Figure 3F). This was similar to the case in mammals, in which A549 cells (human alveolar basal epithelial cells) expressing mOasl2 had a low RIN (6.0), and parental A549 cells (containing OAS1-3) had a relatively high RIN (7.6) (Figure 4H). Similarly, parental DF1 cells, dOASL and oOASL recovery-expression DF1^OASL−/−^ and A549 cells expressing mOasl2 had a low RIN (8.0, 8.2, 8.0, and 7.3, respectively) after being infected by CK/0513 or PR8 viruses. DF1^OASL−/−^ cells did not induce rRNA degradation and had a high RIN (10.0) (Figures 3F and 4I). In summary, these data support that avian OASLs possess 2–5A synthetase activity and activate the OAS/RNase L pathway to inhibit the replication of a range of RNA viruses.

Similar to OASL, RNase L was also known as an ISG (21). Previous studies indicated that exposure of human prostate cancer cells DU145 to physiologic levels of 2–5A (0.1 M) produces a remarkable transcription of ISG (i.e., ISG15) (22). We, therefore, asked whether OASL enhances the activation of OAS/RNase L signaling to prevent viral infection. Interestingly, we found that both dOASL and oOASL significantly increased the expression of *RNase L* and 10 of 16 genes (*IRF1, IRF7, IFN*α, *IFN*β, *IFNAR1, JAK1, STAT1, MX1, PKR*, and *TNF*α) related to IFN signaling in DF1^OASL−/−^ cells induced by the CK/0513 virus (Figures 3G–I). Similarly, mOasl2 significantly enhanced the expression of *RNase L* and six (*IRF3, IFN*α, *IFN*β, *IFIT1, IL8*, and *TNF*α) of 10 tested genes related to IFN signaling in A549 cells after infection by the PR8 virus (Figures 4J–L). These observations supported that enzymatic OASLs enhance OAS/RNase L signaling to degrade viral RNA and magnify IFN signaling to defend against viral infection.

### Duck and Ostrich OASL Reversibly Switch off Their 2–5A Activity Similarly to Human OASL and Mouse Oasl2 When Mutations Were Introduced at Three Conserved D Residues

Previous studies have indicated that three D sites (homologous to D75-D77-D148 in hOAS1) that serve as metal ion ligands are required to synthesize 2–5As (23, 24). To test the effects of these three D residues on the binding affinity for the dsRNA and 2–5A activity of OASLs, we introduced mutations at the homologous sites of the above three D residues to generate dOASL, oOASL, mOasl2, and hOASL mutants. Interestingly, the mutations of the three D residues switched off the 2–5A activity of the OASLs, but seem not to affect their binding affinity for pIC (Figure S4 in Supplementary Material). For example, dOASL-3D*, oOASL-3D*, and mOasl2-3D* lost their 2–5A activity, whereas hOASL-3D recovered 2–5A activity (Figures 4A–C). Detailed analysis indicated that after losing the 2–5A activity, neither dOASL-3D* nor oOASL-3D* induced rRNA degradation or upregulated the expression of *RNase L* or the 16 other genes related to IFN signaling, thus failing to prevent virus infection in DF1 cells and DF1^OASL−/−^ (Figures 4D,E; Figures S5 and S6 in Supplementary Material). However, nonenzymic mOasl2-3D* failed to induce rRNA degradation and upregulate expression of *RNase L*, but significantly inhibited PR8 virus replication in A549 and HeLa (human cervical carcinoma) cells (Figures 4F–L). Moreover, similarly to mOasl2, the expression of hOASL-3D inhibited the PR8 virus replication in both A549 and HeLa cells, induced rRNA degradation when inoculated with either pIC (500 ng/mL) or the PR8 virus (MOI = 1), and increased the expression of *RNase L, IRF3, IFN*α, *IFN*β, *IFIT1, IL8*, and *TNF*α in A549 cells (Figures 4F–K). In summary, these observations suggest that avian and mammalian enzymatic and non-enzymatic OASLs can reversibly change the role in the immune response to viral infection through editing three metal ion ligand sites homologous to D75-D77-D148 in hOAS1.

### Unlike the OLDs of Mouse Oasl2 and Human OASL Mutant (OASL-3D), Those of Duck and Ostrich Cannot Efficiently Activate the OAS/RNase L Pathway

**Figure 5:**
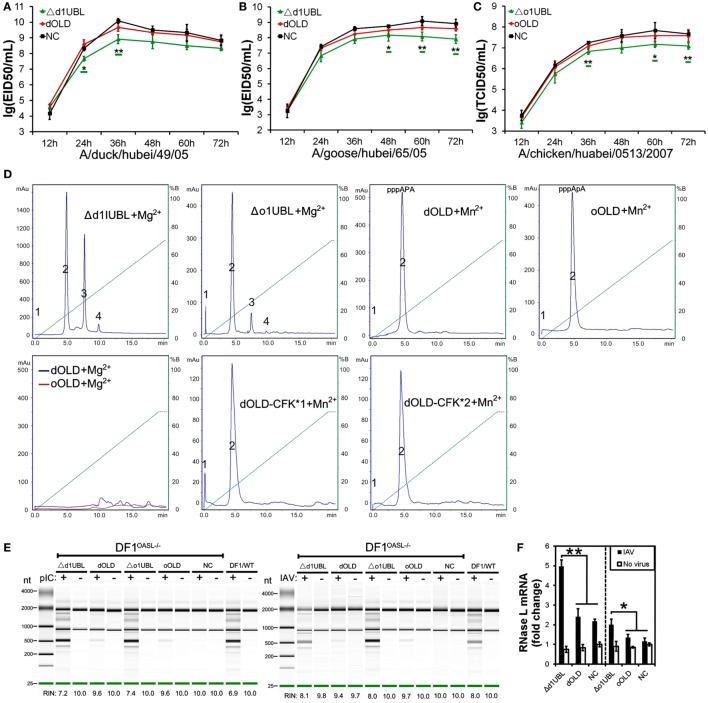
Duck and ostrich OASL activate and magnify the OAS/RNase L pathway in a ubiquitin-like domains (UBL)-dependent manner, while human OASL-3D and mouse Oasl2 do it in a UBL-independent manner. Cells infected with virus were collected at indicated time points to perform EID50 assays or TCID50 assays on MDCK cells. NC is DF1, DF1^OASL−/−^, or A549 cells expressing empty vector. The 2–5A synthetase reaction was treated with alkaline phosphatase and separated using a Mono Q column. “RIN” is the RNA integrity number (*n* = 3). The data are expressed as the mean ± SD. *P < 0.05; **P < 0.01. **(A–C)** Truncations of dOASL and oOASL lacking one UBL, but not lacking both UBLs, significantly inhibited DK/49 **(A)** and GS/65 **(B)** virus replications in DF1 cells or CK/0513 **(C)** virus replications in DF1^OASL−/−^ cells. **(D)** The elution profiles produced by truncations and truncated mutants of four enzymatic OASL lacking one UBL and two UBLs in reaction. **(E)** rRNA cleavage induced by truncations of dOASL and oOASL in DF1^OASL−/−^ cells transfected with pIC for 4 h or CK/0513 virus (multiplicity of infection = 1) for 18 h. **(F)** Truncations of dOASL and oOASL lacking one UBL, but not lacking two UBLs, significantly increased the expression of *RNase L* upon infection with CK/0513 virus in DF1^OASL−/−^ cells.

**Figure 6:**
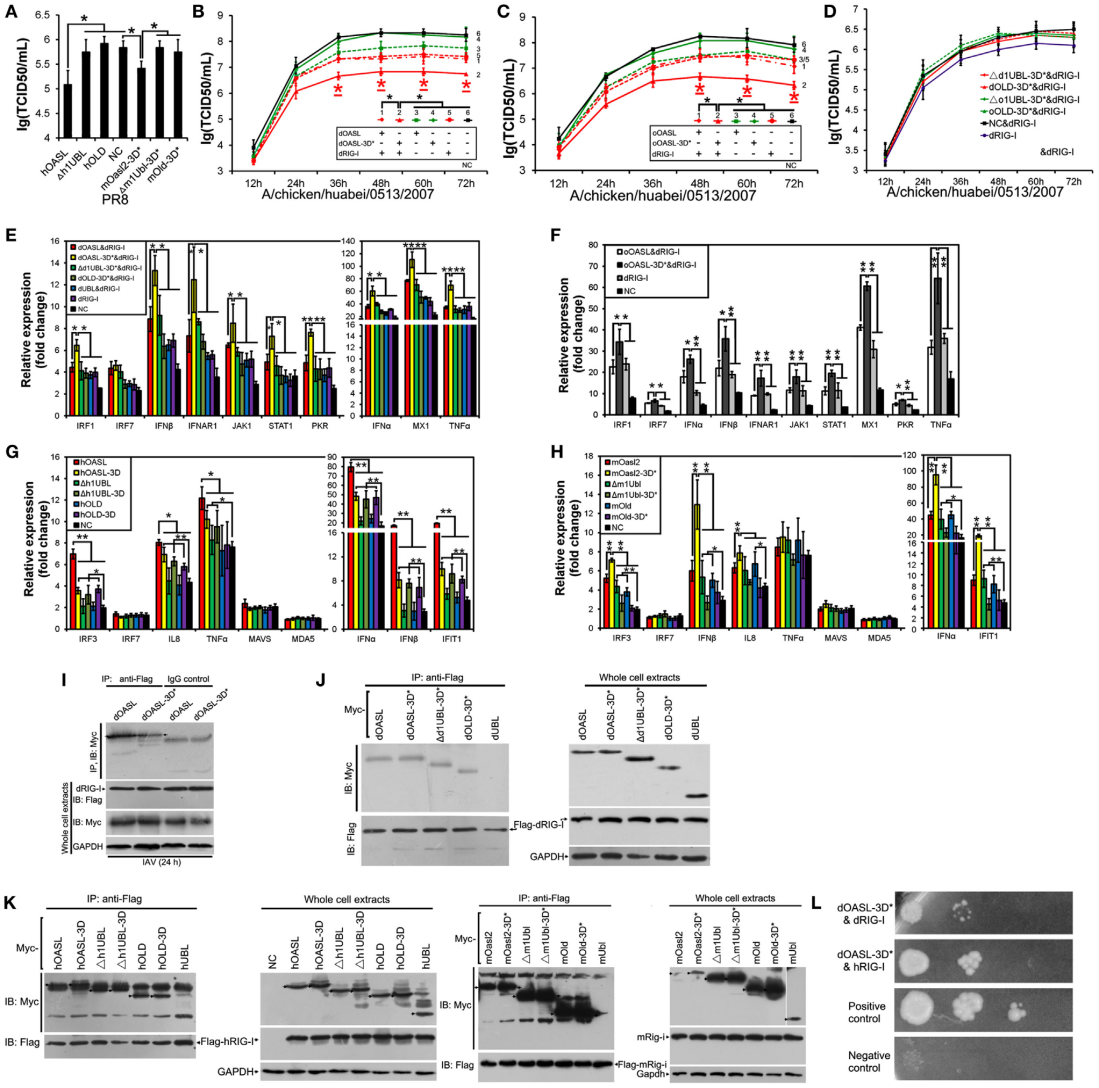
Duck and ostrich OASL-3D* interact with RIG-I and enhance the RIG-I signaling in a ubiquitin-like domains (UBL)-dependent manner, similarly to human OASL and mouse Oasl2-3D*. Cells infected with virus were harvested at the indicated time points and subjected to EID50 or TCID50 assays on MDCK cells. NC is DF1^OASL−/−^ or A549 cells expressing empty vector. Gene expressions in cells were calculated relative mRNA level to that of *GAPDH* and presented as fold change against the corresponding of NC without virus infection (two-tailed Student’s *t*-test, *n* = 3). The data are expressed as the mean ± SD. *P < 0.05; **P < 0.01. **(A,G,H)** hOASL and mOasl2-3D*, but not their truncations, significantly increase expression of six genes downstream of *MAVS* and inhibit PR8 virus replication in A549 cells after infection at 48 h. **(B–F)** Like hOASL and mOasl2-3D*, dOASL-3D* and oOASL-3D*, but not their truncations enhance the antiviral effect of dRIG-I and increased expression of nine genes downstream of *MAVS* in the dRIG-I recovery DF1^OASL−/−^ cells upon CK/0513 infection. **(I–K)** Full length, mutants, and truncations (except UBL) of dOASL, hOASL, and mOasl2 co-precipitate with dRIG-I before and after infected by CK/0513 or PR8 virus. **(L)** Yeast two-hybrid analysis shows that dOASL-3D*, but not dOASL and hOASL, directly interacted with dRIG-I and hRIG-I proteins.

To identify domains that are critical for antiviral activity of OASL proteins, we created Flag-tagged truncations of dOASL, oOASL, hOALS-3D, and mOasl2 that lacked one (Δ1UBL) or two UBLs (OLD) (Figure 1). Interestingly, like the above four enzymatic full length OASLs, two avian and two mammalian truncations lacking one UBLs (Δd1UBL, Δo1UBL, Δh1UBL-3D, and Δm1Ubl) could significantly inhibit one or two H5N1 virus replications through binding dsRNA to synthesize 2–5As and induce rRNA degradation, upregulate expression of *RNase L* and nine (*IRF1, IRF7, IFN*α, *IFN*β, *IFNAR1, JAK1, STAT1, MX1*, and *PKR*) or five (*IRF3, IFN*α, *IFN*β, *IFIT1* and *TNF*α) other genes related to IFN signaling in DF1^OASL−/−^ or A549 cells (Figure 5; Figures S7 and S8 in Supplementary Material). Similarly, two mammalian truncations (hOLD-3D and mOld) lacking both UBLs could bind dsRNA to synthesize 2–5As and induce rRNA degradation, upregulate expression of *RNase L* and four other genes related to IFN signaling (*IRF3, IFN*α, *IFN*β, and *IFIT1*), efficiently activate and enhance the OAS/RNase L to inhibit PR8 virus replication in A549 cells (Figure 6; Figure S8 in Supplementary Material). In sharp contrast, two avian truncations (dOLD and oOLD) lacking both UBLs neither induce rRNA degradation (Figures 5D,E) nor upregulate expression of *RNase L* and genes related to IFN signaling (Figure 5F; Figure S7 in Supplementary Material), thus failing to block one or two H5N1 virus replications in the DF1^OASL−/−^ and DF1 cells (Figures 5A–C). Detailed analysis indicated that dOLD and oOLD bound dsRNA, but failed to synthesize longer oligomers of 2–5Aswith pIC as an activator (Figure 5D; Figure S4 in Supplementary Material). Previous studies demonstrated that a tripeptide motif (CFK) within human *OAS1* and *OAS2* mediates polymerization and affects the synthesis of effective 2–5As (25). We then asked whether mutations in the CFK motif of avian OLD affect their polymerization and further influence their processivity for 2–5A synthesis. We introduced a CFK motif of hOAS1 at a homologous site of dOLD to generate dOLD-CFK*1 and dOLD-CFK*2 substitutions (Figure 1). Unexpectedly, upon Mn^2+^ stimulation and pIC, neither dOLD-CFK*1 nor dOLD-CFK*2 synthesized trimeric or longer oligomers. In contrast, the mOld-CIT* substitution containing the CFK motif of dOASL, also synthesized trimeric or longer oligomers, like mOld did (Figure S8C in Supplementary Material). These results suggest that avian OLDs diverged substantially from both mammalian OAS1 and mammalian OLDs, thus it cannot restore the 2–5A activity of avian OLDs efficiently through the compensation of a conserved CFK motif.

### Duck and Ostrich UBLs in OASLs, but Not Human OASL Mutant (OASL-3D) and Mouse Oasl2 Ones, Bind dsRNA and Are Required to Activate the OAS/RNase L Pathway

Upon discovering that the UBLs of avian OASLs are essential to exert their antiviral activity, we generated Flag-tagged two-tandem UBL truncations of two avian and mammalian OASLs to investigate their functions in the OAS/RNase L pathway (Figure 1). As expected, the two avian (dUBL and oUBL) and two mammalian (hUBL and mUbl) truncations failed to synthesize 2–5As (data not shown), induce rRNA degradation, and change the expression of *RNase L* and genes related to IFN signaling when treated with pIC or infection with H5N1 virus (DK/49, GS/65, CK/0513, or PR8), thus failing to inhibit H5N1 virus replication in DF1 and DF1^OASL−/−^ or A549 cells (Figure S9 in Supplementary Material). These results suggest that the tandem UBLs of avian and mammalian OASLs have no antiviral activity and cannot activate the OAS/RNase L pathway.

Previous studies showed that upon binding dsRNA, OAS synthesizes 2–5As, which in turn activates RNase L to trigger antiviral activity (23, 26, 27). As avian OASLs synthesized short oligomers of 2–5A and failed to activate the OAS/RNase L pathway when their two tandem UBL domains were deleted, we asked whether UBLs improve the 2–5A activity of avian OASLs by enhancing their binding affinity to dsRNA. As expected, dOLD had a lower binding affinity for pIC compared to that of the full protein. However, both mOLD and hOLD-3D showed a comparative level of binding affinity for pIC to their full proteins (Figure S4 in Supplementary Material). Further binding affinity analysis demonstrated that the UBLs of dOASL and oOASLs, but not that of hOASL and mOASL2, bound pIC (Figures S4 and S9 in Supplementary Material). Thus, our data show that the UBL of avian OASLs, but not mammalian OASLs, enhance the binding affinity of OASL to dsRNA and are essential to activate the OAS/RNase L pathway.

### Duck and Ostrich OASL Mutants (OASL-3D*) Enhance RIG-I Signaling in a Similar Manner to Human OASL and Mouse Oasl2 Mutant (Oasl2-3D*)

Recent studies have shown that hOASL reduced a broad range of virus replications through enhancing the RIG-I activation (14, 28, 29). Similarly, hOASL and mOasl2-3D* failed to activate and magnify the OAS/RNase L system, but significantly inhibited PR8 virus replication in A549 cells (Figure 6A). We, therefore, asked whether both avian and mammalian non-enzymatic OASLs or enzymatic OASL mutants utilize the RIG-I pathway to inhibit viral replication. We generated an additional dOASL mutant (dOASL-3K*) through introducing mutations at three conserved positively charged amino acids (K) in the dsRNA-binding groove (Figure 1) (23). As expected, although binding dsRNA, dOASL-3K* together with the other two avian OASL mutants (dOASL-3D* and oOASL-3D*) lacked 2–5A activities and did not activate RNase L to degrade rRNA, failing to prevent virus infection in DF1 and DF1^OASL−/−^ cells (Figures 4D,E; Figures S5 and S6 in Supplementary Material). Because *RIG-I* is absent in chickens, this observation is similar to hOASL, which showed no antiviral activity in the absence of *RIG-I* (17, 29). Interestingly, dOASL-3D* and oOASL-3D*, but not dOASL, oOASL, and dOASL-3K*, significantly enhanced the dRIG-I activation to reduce the CK/0513 virus replication in duck *RIG-I* recovery-expression DF1^OASL−/−^ cells. This observation is similar to the case in mammals, where one non-enzymatic (hOASL) and one enzymatic OASL mutant (mOasl2-3D*) significantly enhanced the RIG-I activation, while their enzymatic OASL proteins (hOASL-3D and mOasl2) and another non-enzymatic mutant (hOASL-R*K*K*) did not (Figures 6A–C; Figures S10 and S11 in Supplementary Material). Further analysis indicated that all truncations of dOASL-3D*, oOASL-3D*, hOASL, and mOasl2-3D* lacking one or two UBLs failed to reduce the CK/0513 and PR8 virus replication in duck *RIG-I* recovery-expression DF1^OASL−/−^ or A549 cells (Figures 6A,D).

RIG-I targets MAVS to initiate downstream signaling, thereby inducing the transcription of type I IFNs and ISGs. We next evaluated whether OASL-3D* induced the expression of RIG-I signaling. Expectedly, both dOASL-3D* and oOASL-3D*, but not dOASL, oOASL, and dOASL-3K*, significantly increased the expression of nine genes (*IRF1, IFN*α, *IFN*β, *IFNAR1, JAK1, STAT1, MX1, PKR*, and *TNF*α) downstream of *MAVS* after infecting by CK/0513 virus (Figures 6E,F; Figure S10 in Supplementary Material). Whereas neither avian OASL nor their mutants affected the expression of *MAVS, MDA5*, and *LGP2* in duck *RIG-I* recovery-expression DF1^OASL−/−^ cells (data not shown). Similarly, two mammalian non-enzymatic OASL proteins (hOASL and mOasl2-3D*), but not their enzymatic OASL proteins, significantly increased the expression of five genes in the RIG-I signaling pathway (*IRF3, IFN*α, *IFN*β, *IFIT1*, and *IL8*) in A549 cells infected with the PR8 virus (Figures 6G,H; Figure S11 in Supplementary Material). We further compared the gene expression of the RIG-I pathway in duck *RIG-I* recovery-expression DF1^OASL−/−^ cells and A549 cells that expressed truncations lacking one or two UBLs of dOASL-3D*, hOASL, or mOasl2-3D*. This effort found that none of them changed the expression of the above tested genes of the RIG-I signaling pathway with or without IAV infection (Figures 6E–H), further supporting that OLD and the two UBLs of avian and mammalian OASLs are essential for magnifying the RIG-I pathway.

We then investigated the interaction between RIG-I and OASL and found that both dOASL and dOASL-3D* co-precipitated with dRIG-I in DF1 cells before and after infection with the CK/0513 virus (Figures 6I,J). Similarly, we observed that hOASL, mOasl2, and their mutants (hOASL-3D and mOasl2-3D*) co-precipitated with human or mouse RIG-I in 293T cells (Figure 6K). Thus, the mutation of the three conserved D residues did not affect the interaction between OASL and RIG-I. Detailed analysis indicated that UBL-deleted OASL (Δh1UBL, hOLD, Δm1Ubl, and mOld) and their corresponding mutants (Δd1UBL-3D*, dOLD-3D*, Δh1UBL-3D, hOLD-3D, Δm1Ubl-3D*, and mOld-3D*) also interacted with RIG-I, whereas the UBL of OASL alone (dUBL, hUBL, and mUBL) did not (Figures 6J,K). This observation, combined with that truncations of avian and mammalian non-enzymatic OASL proteins, did not prevent against virus infection, indicates that the OLD domain of OASL is sufficient to mediate the interaction between OASL and RIG-I but insufficient to enhance RIG-I signaling. We further investigated the module of this interaction using a yeast two-hybrid system. This effort found that among the above OASLs and their mutants from ducks and humans, only dOASL-3D* directly interacted with intact duck and human RIG-I protein (Figure 6L).

## Discussion

**Figure 7:**
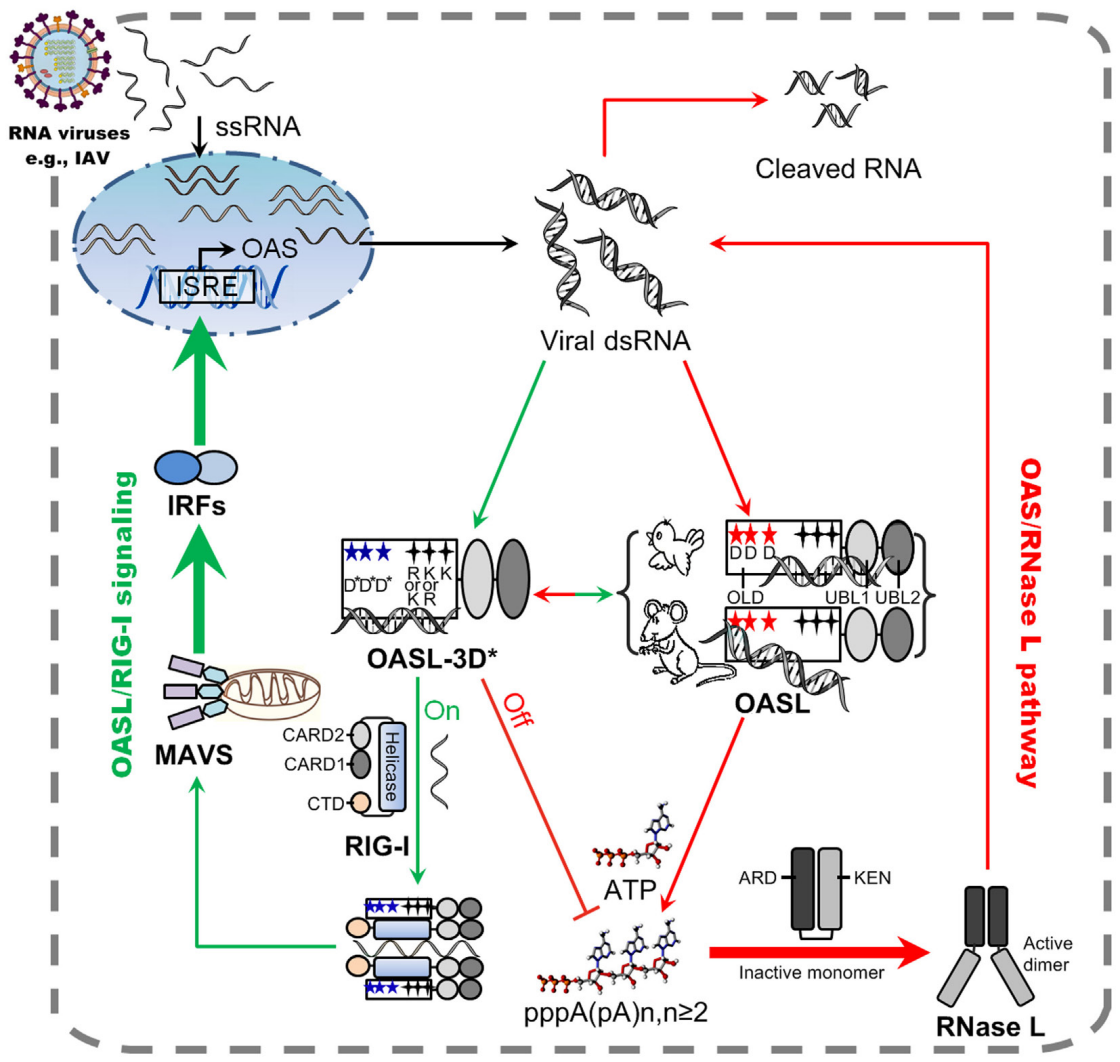
Model of avian and mammalian OASL involvement in RNase L and RIG-I signaling. As a sensor, ancient and enzymatic OASL in birds and mammals contain three aspartic acids (D), which are homologous to D75-D77-D148 in hOAS1 and required to synthesize 2–5As, and three key positively charged amino acids (R/K-K/R-K), which are homologous to R195-K199-K205 in human 2′–5′-oligoadenylate synthetase 1 (OAS1). Both avian and mammalian OASLs develop the potential ability to switch the OAS/RNase L and OASL/RIG-I signaling. In natural, avian OASL activates and magnifies the OAS/RNase L pathway to exert antiviral activity in an UBL-dependent manner, where both its OAS-like domain (OLD) and UBL domains bind dsRNA. While some enzymatic mammalian OASLs activate and magnify the OAS/RNase L pathway to exert antiviral activity in an UBL-independent manner, where only their OLD domains bind dsRNA and synthesizes 2–5A like human OAS1. Some non-enzymatic mammalian OASLs lose 2–5A activity, switch to activate, and enhance RIG-I signaling to inhibit the replications of RNA viruses in a UBL-dependent manner when their three conserved D sites are mutated. Such natural switching between the OAS/RNase L and OASL/RIG-I using OASL molecules were recently, thus, both avian and mammalian OASL may reversibly to activate and magnify one of the above signaling when we introduce mutations at three conserved D sites. Five-pointed star (red): D residue; Five-pointed star (blue): D sites serving as metal ion ligands were mutated to A residue, or E81, E83, and T152 site in hOASL; Four-pointed star (black): three key positively charged amino acids; Four-pointed star (green): three key positively charged amino acids (RKK or KKK) were mutated to E. Abbreviations: OLD, OAS-like domain; UBL, ubiquitin-like domain; ARD, ankyrin repeat domain; KEN, kinase-extension-nuclease domain; CARD, caspase activation and recruitment domain; CTD, carboxy-terminal domain; ISRE, interferon-stimulated response element; MAVS, mitochondrial antiviral signaling protein; IRF, IFN-regulatory factor; ssRNA, single-stranded RNA; dsRNA, double-stranded RNA.

Here, we first demonstrated that, after stimulation by dsRNA, two divergent avian OASLs (dOASL and oOASL) activated RNase L to induce rRNA degradation using their 2–5A products like mammalian enzymatic OASL (mOasl2) (Figures 3 and 7). We find that three conserved D residues were crucial to adaptively reversible switching between enzymatic and non-enzymatic OASL, where mutants of one mammalian (mOasl2) and two avian enzymatic OASL (dOASL and oOASL) lose their 2–5A activity and mutant of one mammalian non-enzymatic OASL (hOASL-3D) recover its 2–5A activity. We also found that two avian (dOASL and oOASL) and two mammalian (hOASL-3D and mOasl2) enzymatic OASLs significantly increased the expression of *RNase L* and 10 (*IRF1, IRF7, IFN*α, *IFN*β, *IFNAR1, JAK1, STAT1, MX1, PKR*, and *TNF*α) or six (*IRF3, IFN*α, *IFN*β, *IFIT1, IL8*, and *TNF*α) genes related to IFN signaling in DF1^OASL−/−^ cells or in A549 cells after infection with the H5N1 (CK/0513 or PR8) virus (Figures 3 and 4). This is consistent with the fact that 2–5A induces the gene expression of several ISGs (*P56, P54, IL8*, and *ISG15*) in DU145 prostate cancer cells and HeLa cells (22). Therefore, our observations support to the theory that the ancient OASL of birds and mammals possessed 2–5A activity and executed their antiviral activity through activating and magnifying the OAS/RNase L pathway and enhancing IFN signaling. Moreover, our functional analyses strengthen the idea that species-specific adaptations appear to accelerate the functional divergence of the OASL molecules. For example, avian OASLs developed a UBL-dependent manner to activate and magnify the RNase L system and IFN signaling to block virus replication. In this UBL-dependent model, the OLDs of avian OASLs bind dsRNA and synthesize 2–5As, but cannot activate RNase L to degrade rRNA and inhibit virus replication (Figures 5 and 7). The UBLs of avian OASLs bind dsRNA like their OLDs, and truncations of avian OASLs lacking both UBLs showed weak binding affinity for dsRNA (Figures S4 and S9 in Supplementary Material). These observations, together with the abnormality in the 2–5A synthesis activity of dOLD and oOLD (Figure 5), supported the idea that UBLs of avian OASLs have been optimized to bind dsRNA using their enriched positive amino acids (A, H, and L). This optimization, in return, improved avian OASLs’ polymerization and processivity for 2–5A synthesis and contributed to the activation and magnification of RNase L signaling. We then hypothesized that avian OASLs could bind dsRNA with different lengths and showed antiviral activity to a range of viruses. This hypothesis was supported by our data. Such as, both dOASL and oOASL synthesized 2–5As at high level after induction with either low or high weight pIC and significantly inhibited the replication of two strains of double-stranded (IBDV/B87 and REOV/Z97/C10) and four strains of negative single-stranded (DK/49, GS/65, CK/0513, and NDV/La Sota) RNA viruses (Figures 2 and 3). Additionally, dOASL protected against a strain of positive single-stranded (FMDV/O/Mya) RNA virus infection in IBRS2 cells (Figure 2). For mammalian enzymatic OASLs (hOASL-3D and mOasl2), they activate and magnify the RNase L system and IFN signaling to block virus replication in a UBL-independent manner like hOAS1 (30). In this case, both hOLD-3D and mOld could activate RNase L to decay rRNA with their 2–5A products, magnify the RNase L and IFN signaling, and inhibit the PR8 virus replication (Figure 5). Since the UBLs of hOASL-3D and mOasl2 did not bind dsRNA, they appear to be functionally redundant for the activation and magnification of the OAS/RNase L system (Figure S4 in Supplementary Material).

Upon detecting viral dsRNA, mammalian enzymatic OASLs activate RNase L to induce the endonucleolytic cleavage of viral and cellular ssRNAs using their 2–5A products (21, 24, 25), while some mammalian non-enzymatic OASLs (such as human OASL) cannot synthesize 2–5A and activate RNase L to degrade viral and cellular ssRNAs, but can mediate RIG-I activation by mimicking polyubiquitin to inhibit virus replication (14, 29). Interestingly, we found that such natural switching between the OAS/RNase L and OASL/RIG-I signaling are reversible and mediated by three crucial D residues in birds and mammals. When mutations were introduced at three conserved D residues homologous to D75-D77-D148 of hOAS1, two avian (dOASL-3D* and oOASL-3D*) and one mammalian (mOasl2-3D*) OASL mutants lost 2–5A activity and changed to enhance the RIG-I signaling-like hOASL did (Figures 4 and 7; Figures S9 and S11 in Supplementary Material). In contrast, one mammalian OASL mutant (hOASL-3D) restored 2–5A activity, activated and magnified the OAS/RNase L pathway like two avian and one mammalian enzymatic OASL did (dOASL, oOASL, and mOsl2) (Figures 3 and 4). However, when mutations were introduced at three conserved K (or R) residues homologous to R195-K199-K205 of hOAS1 in the dsRNA-binding groove (Figure 1) (23, 31), dOASL and hOASL mutants (dOASL-3K* and hOASL-R*K*K*) activated and magnified neither the OAS/RNase L nor the RIG-I pathway (Figures S6 and S10 in Supplementary Material). These observations suggested that three conserved D residues in the OLD of OASL acted as a switch for the adaptive exchange between the OAS/RNase L and RIG-I pathways, while three positively charged K (or R) residues in the dsRNA-binding groove of OASLs are essential to both the OAS/RNase L and RIG-I pathways. Moreover, we found that two tandem UBL domains were required for avian and mammalian non-enzymatic OASLs (dOASL-3D*, oOASL-3D*, hOASL, and mOasl2-3D*) to enhance and magnify the RIG-I pathway, even they interacted with RIG-I in a UBL-independent manner (Figure 6; Figure S10 in Supplementary Material). The UBL of hOASL was reported to mediate its specific interaction with methyl CpG-binding protein 1 (MBD1), which is an ISG and functions as a transcriptional repressor (32). However, manually querying the MBD1 repertoire against the non-redundant database in NCBI and examining avian genome assemblies in Ensembl (release 87) indicated that MBD1 appears to be absent in birds (data not shown). Thus, avian OASLs may not enhance the RNase L, IFN, and RIG-I signaling by binding the MBD1 protein.

Mouse *Oasl1* specifically suppresses *IRF7* translation by binding to a double stem-loop structure in its 5’UTR, thus negatively regulates IFN during viral infection (15). We found that, unlike mOasl1, dOASL did not suppress *IRF7* translation through binding its 5’UTR (Figure S12 in Supplementary Material). This finding is consistent with our observation that avian OASLs slightly or significantly increase the expression of *IRF7* and then significantly upregulate the expression of *IFN*α and *IFN*β in DF1^OASL−/−^cells infected by the CK/0513 virus (Figure 3). Birds, therefore, do not present a negative feedback pathway for the IFN response through OASL like some mammals (i.e., mouse). In the future, our ability to describe the structure of OASL protein using cryo-electron microscopy and to identify target proteins or RNAs through immunoprecipitation combined with high throughput sequencing will extend our knowledge about the role of OASL in the activation and regulation of *RNase L* and IFN signaling as well as RIG-I signaling.

## Materials and Methods

### Facility and Ethics Statement

Studies of one H1N1 (A/Puerto Rico/8/34, PR8) and three H5N1 viruses (A/duck/Hubei/49/05, DK/49; A/goose/Hubei/65/05, GS/65; A chicken/huabei/0513/2007, CK/0513) were conducted in a biosecurity level 3+ laboratory approved by Chinese Ministry of Agriculture or China Agricultural University. The NDV/La Sota, IBDV/B87, REOV/Z97/C10, FPV/CVCC/AV1003, PRV/Henan/2014, and FMDV/O/Mya viruses were maintained in a biosecurity level 2+ laboratory approved by China Institute of Veterinary Drug Control or Lanzhou Veterinary Research Institute. The age of 10 days (10-day-old) chicken embryos were obtained from Hualan Chen’s lab, and chicken embryos studies were approved by the Review Board of Harbin Veterinary Research Institute, Chinese Academy of Agricultural Sciences.

### Cell Culture and Viral Infections

DF1 (Chicken embryonic fibroblasts cells), 293T (human embryonic kidney 293T cells), A549 (human alveolar basal epithelial cells), HeLa (human cervical carcinoma cells), Vero (African green monkey kidney cells), MDCK (Madin-Darby canine kidney cells), BHK21 (baby hamster kidney fibroblast cells), and C2C12 (murine myoblast cells) were purchased from American Type Culture Collection. PK15 (porcine kidney epithelial cells) and IBRS2 (porcine kidney cells) were obtained from the Cell Resource Center, Peking Union Medical College. All the above cells were maintained in Dulbecco’s modified Eagle’s medium supplemented with 10% fetal bovine serum (FBS; Gibco, Carlsbad, CA, USA) in an atmosphere of 5% CO_2_ at 37°C. Viruses were propagated in 10-day-old chicken embryos. Three samples of cells inoculated with a multiplicity of infection (MOI) of 0.1, 0.01, or 0.001 with one of the above virus after 6, 12, 18, 24, 36, 48, 60, and/or 72 h were collected to monitor virus replication. Titers were calculated by the egg infectious dose (EID50) individuals using the Reed and Muench method (DK/49, GS/65, and NDV), monitored tissue culture infective dose (TCID50) of the cytopathic effect of end-point dilutions (CK/0513, PR8, IBDV, REOV, PRV, and FMDV), or quantified through quantitative PCR with the primer listed in Table S1 in Supplementary Material (FPV).

### Construction and Expression of Recombinant Plasmids

The coding sequences of dOASL, dRIG-I, oOASL, and mOasl2 were amplified from whole duck lungs infected with the DK/49 virus, an whole ostrich spleen cDNA library or C2C12 cells according their gene sequences (KC869660.1, XM_009673088, and NM_011854) using the primers in Table S2 in Supplementary Material (33, 34). Truncations and mutants of dOASL, oOASL, hOASL, and mOasl2 were generated using specific PCR and site-directed mutagenesis, respectively (Tables S2–S4 in Supplementary Material). Full-length, truncated and mutant dOASL, dRIG-I, oOASL, hRIG-I, and mRig-i were cloned individually into the piggyBac (containing a Flag-tag) (35), pCMV-Myc, or pCMV-HA vectors (Clontech, Mountain View, CA, USA) (Tables S2–S4 in Supplementary Material) and were transfected to cells using Lipofectamine 3000 (Thermo Fisher, Carlsbad, CA, USA). Gene expression in cells was examined by Western blotting using the anti-Flag, anti-Myc or anti-HA antibody (1:1,000).

### Establishment of OASL-Deficient DF1 Cells

DF1 cells were co-transfected with Cas9 nuclease and sgRNA plasmids, subjected to trypsin digestion and limiting dilutions using methods similarly to those applied to human cells (19). Clones with large fragment deletion and biallelic mutations in targeted genes were selected through PCR using gene-specific primers covering the region targeted by sgRNA, and they were subsequently confirmed by sequencing (Tables S5 and S6 in Supplementary Material).

### Protein Purification, Detection of 2–5A Activity, and dsRNA-Binding Affinity

Full length, truncated and mutant of OASLs with an affinity His-tag were cloned into the pET-28a(+) vector using the primers in Tables S3 and S4 in Supplementary Material, and transformed into *Escherichia coli* BL21 Codon Plus RIPL (TransGen, Beijing, China) cells. Bacteria were induced to express OASL protein by the addition of 0.5–1.0 mM isopropyl-β-d-thiogalactoside overnight at 18–25°C and were lysed in 25 mL of buffer A using an AvestinEmulsiFlex C3 (Avestin, Ottawa, ON, Canada). Thereafter, the recombinant proteins were isolated using Ni^2+^-NTA affinity column chromatography and further purified with a Heparin HiTrap (5 mL) column (GE Healthcare, Uppsala, Sweden). The 2–5A activity of the recombinant proteins was detected using Mono Q purification, similarly to that applied to mOasl1 and mOasl2 (9). Binding affinity of OASL proteins to dsRNA were evaluated using an Octet RED platform (ForteBio, Menlo Park, CA, USA). The affinities were derived by fitting the kinetic data to a 1:1 Langmuir binding model utilizing global fitting algorithms (36). The dissociation constants K_D_, K on (association rate), and K off (dissociation rate) were determined by fitting the binding chromatogram data with the Octet User Software (version 3.1).

### Co-Immunoprecipitation and Immunoprecipitation

Cells were transfected with equal amounts of Flag- and/or Myc-tagged recombinant plasmids (8 µg) or 3 × Flag-tagged recombinant plasmids (15 µg) using Lipofectamine 3000 reagents (Thermo Fisher, Carlsbad, CA, USA) and lysed in IP lysis buffer (Beyotime, Beijing, China) containing a final concentration of 1 mM PMSF (Beyotime, Beijing, China) after transfection for 24 h. The lysate was cleared using protein A + G agarose (Beyotime, Beijing, China) and specific IgG for 3 h at 4°C and then incubated with anti-Flag immunoglobulin (1:1,000; Abcam, Cambridge, MA, USA) and protein A + G agarose overnight at 4°C. After that, the immunoprecipitated proteins were analyzed using SDS-PAGE following silver staining and a liquid chromatography-mass spectrometry (LC-MS, Q-TOF) assay in BGI-Beijing or Western blotting using a mouse monoclonal c-Myc antibody (1:1,000; Clontech, Mountain View, CA, USA).

### Yeast Two-Hybrid Analysis

Full-length RIG-I were cloned into the bait vector pGBKT7 to create fusion proteins with the Gal4 DNA-binding domain and OASL proteins were individually cloned into the prey vector pGADT7. Both recombinant bait and prey vectors were transformed into the *S*. *cerevisiae* host strain AH109 using the lithium acetate/polyethylene glycol method (Clontech, Mountain View, CA, USA). Positive clones in which the expressed prey protein interacted with the bait protein were selected on minimal double-dropout medium (lacking L and W), assessed on triple selection plates (lacking L, W, and H), and patched onto plates with higher stringency quadruple-dropout medium (without L, W, H, and Adenine). Primers used for the yeast two-hybrid analysis are listed in Table S7 in Supplementary Material.

### Quantitative RT-PCR and rRNA Cleavage Assay

Total RNA was isolated from cells using the RNeasy Plus Mini Kit (Qiagen, Hilden, Germany) or TRIzol (Invitrogen, Rockville, MD, USA) reagent. Then, RNA was DNase-treated (Qiagen, Hilden, Germany) and resolved on RNA chips using an Agilent 2100 BioAnalyzer. RNA integrity was assessed by RIN score (37). cDNA was synthesized with Oligo(dT)18 primer or gene-specific primers using the Promega Improm-II reverse transcriptase (Promega, Madison, WI, USA) and used to examine gene expression using primers in Table S1 in Supplementary Material through normalizing the corresponding expression of the *GAPDH* reference gene. Gene differential expression between samples was calculated using 2^−ΔΔCT^ method (38).

## Author Notes

All institutional and national guidelines for the care and use of laboratory animals were followed.

## Author Contributions

YH, NL, and JL designed the project. ER, XW, and JH constructed recombinant plasmids, generated OASL-positive and OASL-deficient cells, performed rRNA degradation, and carried out quantitative RT-PCR experiments. ER and XW purified recombinant proteins, detected 2-5A activity, carried out RNA binding affinity, and performed other biochemical studies. ER, HC, CY, JL, XW, ZW, WL, XC, HZ, JP, and HS performed virus infection and replication experiments. ER and YH wrote the manuscript. YH, JL, DB, JS, and ER revised the manuscript.

## Conflict of Interest Statement

The authors declare that the research was conducted in the absence of any commercial or financial relationships that could be construed as a potential conflict of interest.
