# Supplementary material for: Molecular Mechanisms for the Adaptive Switching Between the OAS/RNase L and OASL/RIG-I Pathways in Birds and Mammals
Source: Front Immunol. 2018 Jun 20;9:1398. doi: 10.3389/fimmu.2018.01398 (PMC6019448; doi:10.3389/fimmu.2018.01398)
Supplement: Supplementary file 1 [file data_sheet_1.docx]

**Supplementary File**


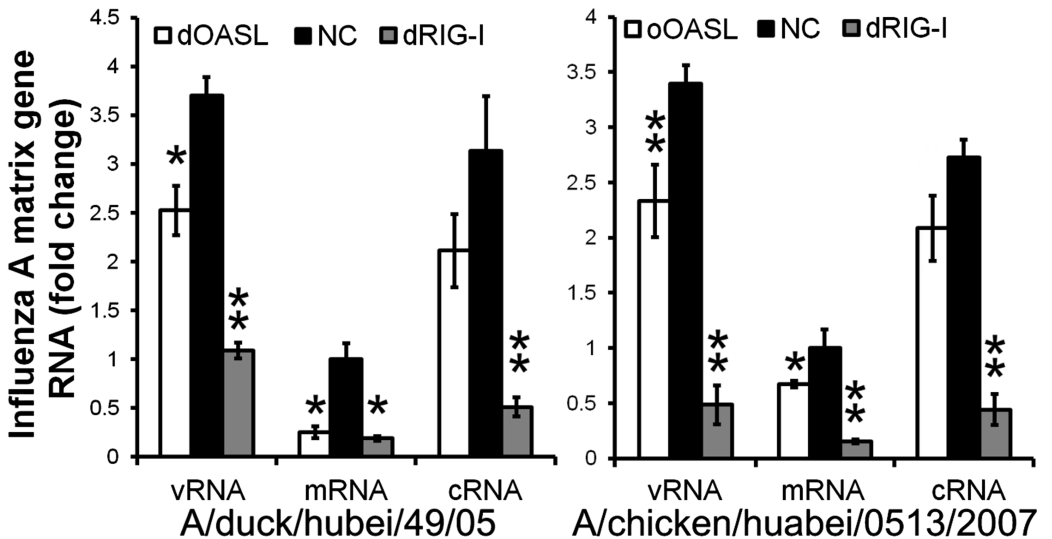


**Fig. S1 Transcription and replication of influenza A matrix gene in stable or transfected DF1 cells.** Cells infected with the DK/49 or CK/0513 virus for 24 h were collected (n = 3). The vRNA, mRNA and cRNA levels of matrix gene were normalized against *GAPDH*. The data are expressed as the mean ± S.D. **P*<0.05; ***P*<0.01.


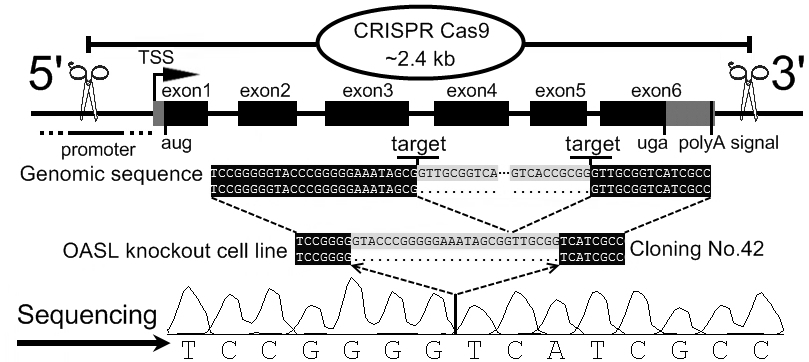


**Fig. S2 Construction of chicken OASL knockout DF1 cells.** Schematic representation of chicken OASL knockout DF1cells using the CRISPR/Cas9-mediated genome editing method. We used two target sites to delete the complete chicken OASL gene from genome sequence. Clones deleted large fragment deletion and biallelic mutation in targeted genes were selected through PCR using gene-specific primers covering the region targeted by sgRNA, and subsequently confirmed by sequencing. TSS, transcription start site. aug and uga represents start and stop codon, respectively.


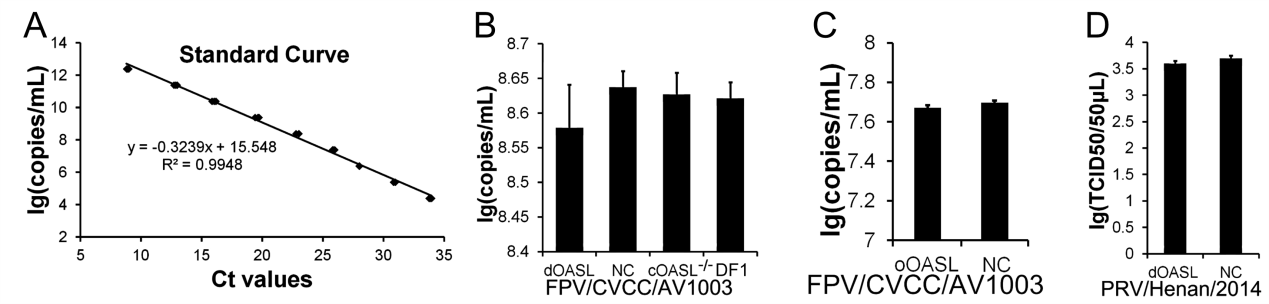


**Fig. S3 Avian OASL doesn’t inhibit the replication of FPV and PRV virus in DF1 cells.** (A) FPV titers were determined by qPCR post infection at 120 h, the standard curve revealed the linear relationship between Ct (cycle threshold) and FPV copies (R^2^ = 0.9948). (B) Duck OASL did not prevent against FPV (MOI=0.1) in DF1 cells and deletion of chicken OASL in DF1 cells (DF1^OASL−/−^) did not enhance FPV replication. NC is chicken DF1 cell expressing empty vector. (C) Ostrich OASL did not prevent against FPV replication in DF1 cells. (D) Duck OASL did not prevent against PRV (MOI=0.01) in PK15 cells. PRV titers were determined by TCID50 assays on PK15 cells at 24 h post infection. The data are expressed as the mean ± S.D.


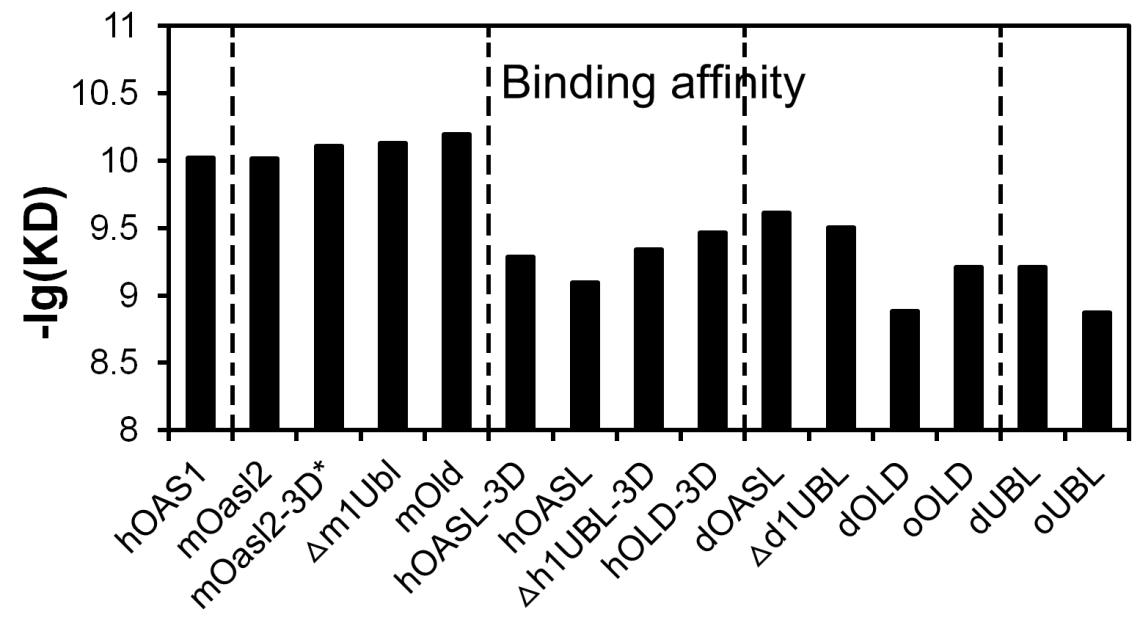


**Fig. S4 The binding affinity to poly(I:C) of avian and mammalian OASL recombinant proteins.** Binding affinity assay were estimated using an Octet RED platform.


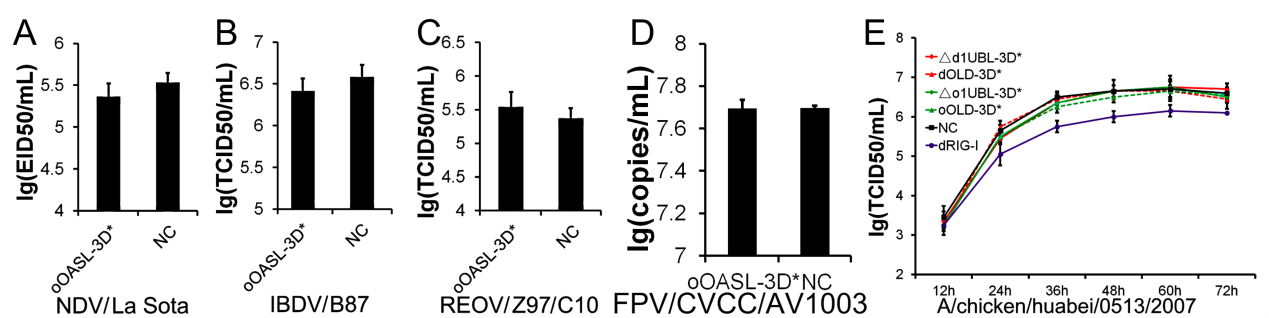


**Fig. S5 Ostrich OASL mutant (OASL-3D*) and truncations of duck and ostrich OASL mutants (OASL-3D*) lacking one or two UBLs do not affect viral replication.** (A-D) DF1 cells expressing ostrich OASL-3D* did not prevent against NDV (A), IBDV (B), REOV (C) and FPV (D) virus. (E) Duck and ostrich Δ1UBL-3D* and OLD-3D* truncations did not affect the replication of CK/0513 virus in DF1^OASL-/-^ cells. The data are expressed as the mean ± S.D.


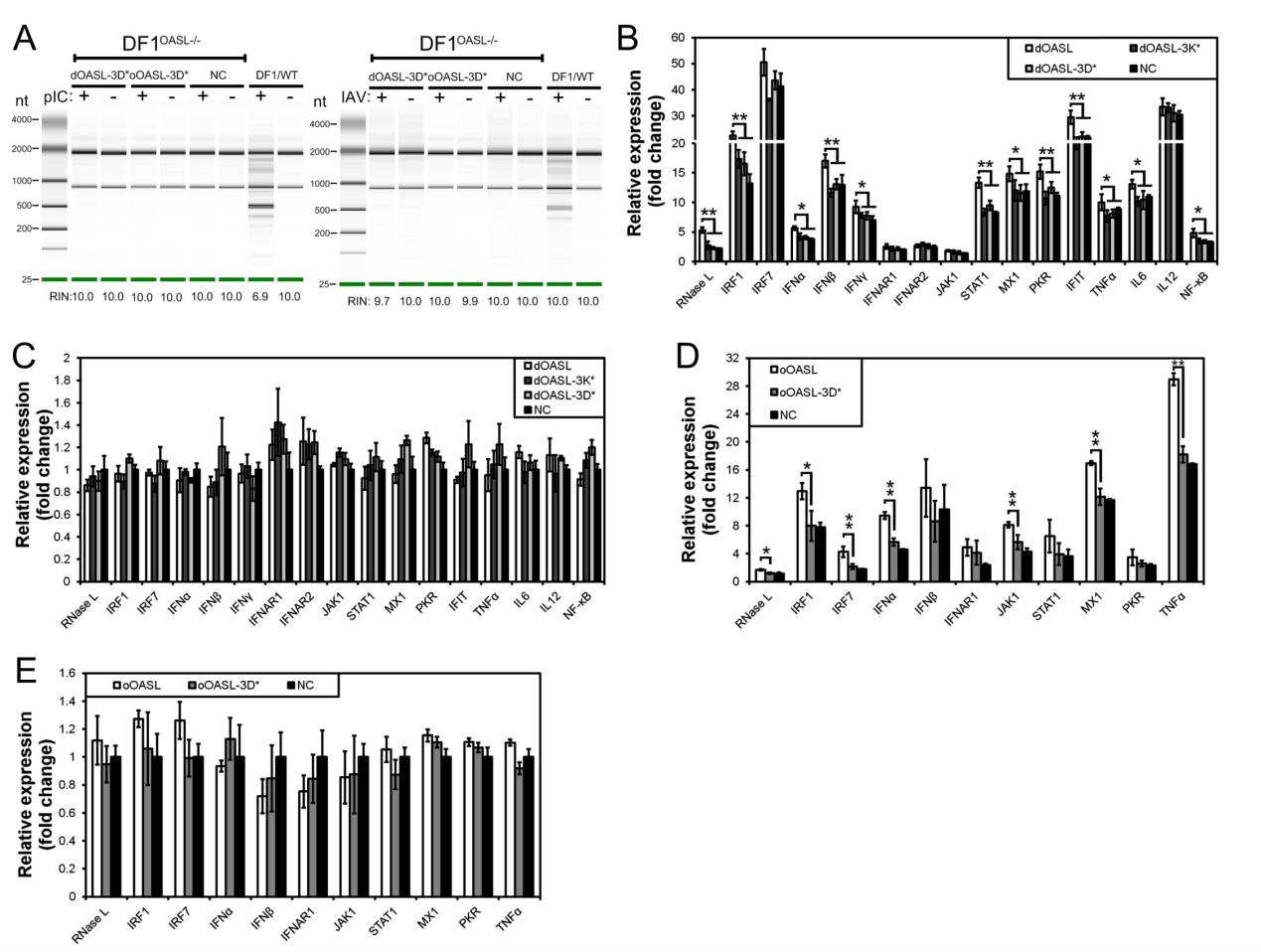


**Fig. S6 Duck and ostrich OASL-3D*/3K* do not induce rRNA degradation and change the expression of RNase L and several genes related to IFN signaling in DF1^OASL−/−^ cells.** Expression of above genes in cells were calculated relative mRNA level to that of *GAPDH* and presented as fold change against the corresponding of DF1^OASL−/−^ cells expressing empty vector (NC) without infection of CK/0513 virus. **P*<0.05; ***P*<0.01 (two–tailed Student’s test, n = 3 for each group). (A) Neither duck nor ostrich OASL-3D* protein induces rRNA cleavage in DF1^OASL−/−^ cells upon co-transfection with pIC (left) or infection with CK/0513 (right) virus. (B-C) Duck OASL-3D*/3K* do not significantly change the expression of *RNase L* and 16 genes related to IFN signaling in DF1^OASL−/−^ cells with (B) or without (C) infection of CK/0513 virus (MOI = 0.001). (D-E) Ostrich OASL-3D* do not change the expression of RNase L and 10 genes related to IFN signaling in DF1^OASL-/-^ cells with (D) or without (E) infection of CK/0513 virus.


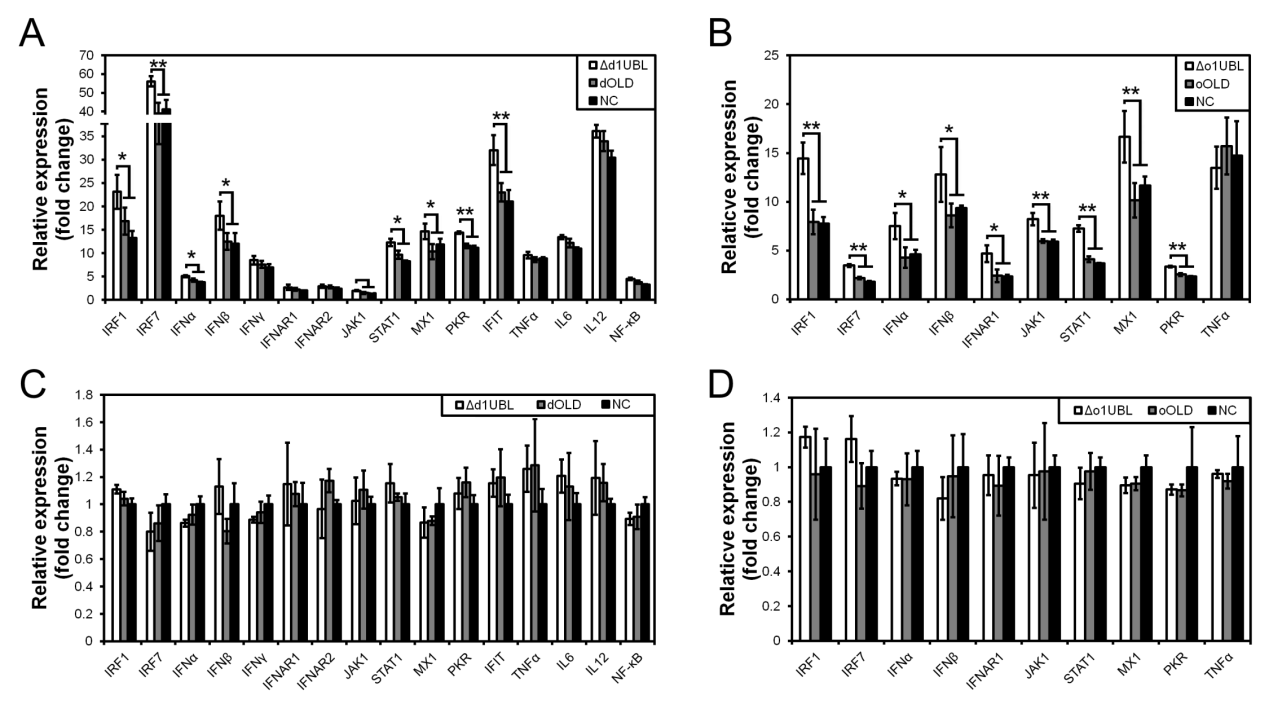


**Fig. S7 Duck and ostrich Δ1UBL, but not OLD truncation, significantly change the expression of several genes related to IFN signaling in DF1^OASL−/−^ cells.** Expression of above genes in cells were calculated relative mRNA level to that of *GAPDH* and presented as fold change against the corresponding of DF1^OASL−/−^ cells expressing empty vector (NC) without infection of CK/0513 virus. **P*<0.05; ***P*<0.01 (two–tailed Student’s test, n = 3 for each group). (A-B) DF1^OASL−/−^ cells expressing one truncations of Δd1UBL, dOLD, Δo1UBL and oOLD with infection of CK/0513 virus (MOI = 0.001). (C-D) DF1^OASL−/−^ cells expressing one truncations of Δd1UBL, dOLD, Δo1UBL and oOLD without infection of CK/0513 virus.


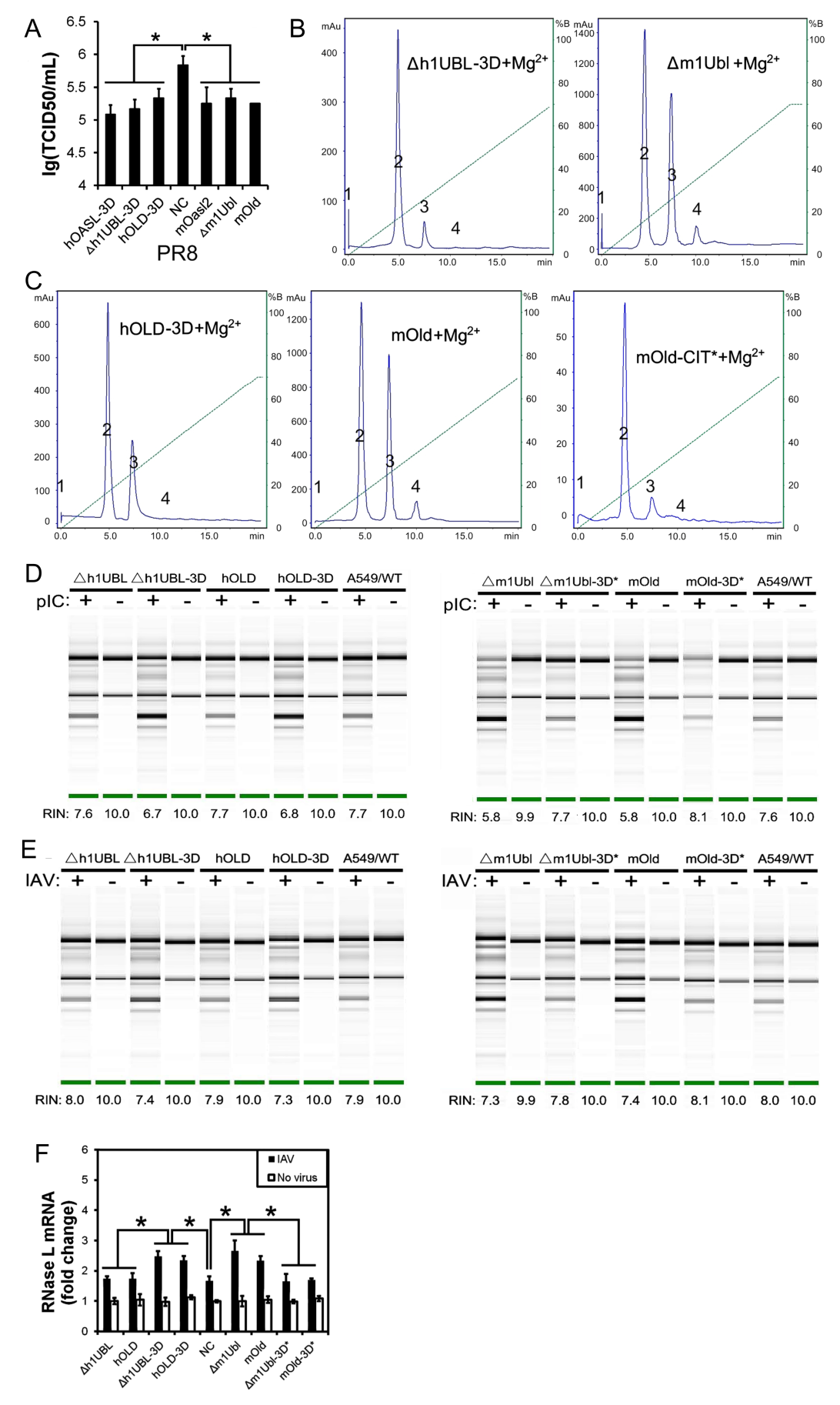


**Fig. S8 Human OASL-3D and mouse Oasl2 activate and magnify the OAS/RNase L pathway in a UBL-independent manner.** Cells infected with virus were collected at 48 h to perform TCID50 assays on MDCK cells. NC is A549 cells expressing empty vector. The 2-5A synthetase reaction was treated with alkaline phosphatase and separated using a Mono Q column. “RIN” is the RNA integrity number (n = 3). The data are expressed as the mean ± S.D. **P*<0.05; ***P*<0.01. (A) Truncations of hOASL-3D and mOasl2 lacking one and both UBL significantly inhibited PR8 virus replication like their full proteins in A549 cells. (B-C) The elution profiles produced by truncations and truncated mutants of four enzymatic OASL lacking one UBL and two UBLs in reaction. (D-E) rRNA cleavage induced by truncations of hOASL-3D and mOasl2 in A549 cells transfected with pIC for 4 h or PR8 virus (MOI = 1) for 18 h. (F) Truncations of hOASL-3D and mOasl2 lacking one and two UBL significantly increased the expression of *RNase L* upon infection with PR8 virus in A549 cells.


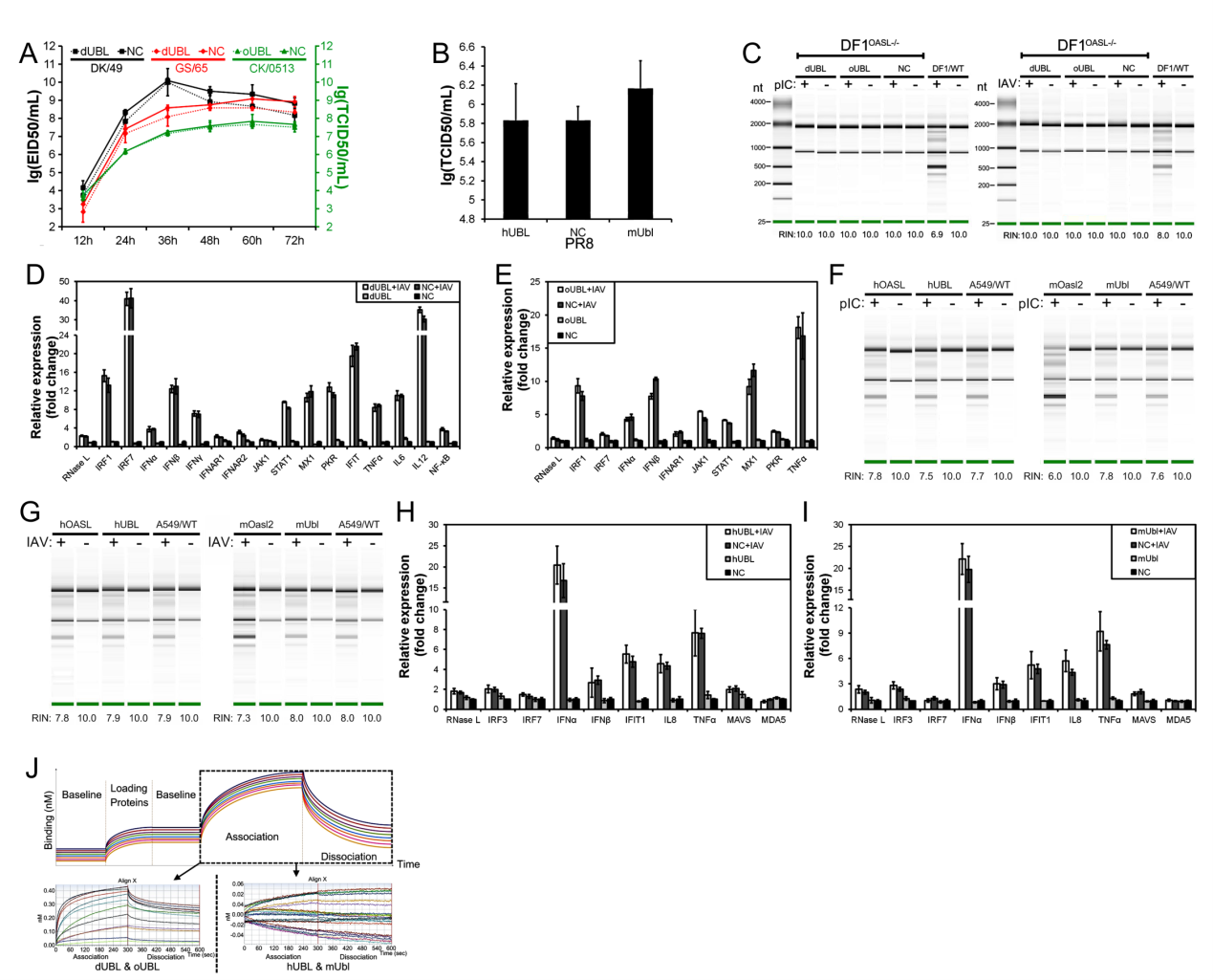


**Fig. S9 UBLs of OASLs do not affect viral replication and induce rRNA degradation, avian but not mammalian ones improve the binding affinity of OASL to dsRNA.** Cells infected with virus were collected at indicated time points to perform EID50 assays or TCID50 assays on MDCK cells. NC is DF1 and DF1^OASL-/-^ or A549 cells expressing empty vector. “RIN” is the RNA integrity number. Gene expressions in cells were calculated relative mRNA level to that of *GAPDH* and presented as fold change against the corresponding of NC without virus infection (n = 3). The data are expressed as the mean ± S.D. (A) UBL of duck and ostrich OASL did not prevent against the DK/49 and GS/65in DF1 cells and CK/0513 virusin DF1^OASL-/-^ cells. (B) UBLs of human OASL and mouse Oasl2 do not inhibit replication of PR8 virus in A549 cells. (C) Neither duck nor ostrich UBL truncations induces rRNA cleavage in DF1^OASL−/−^ cells upon co-transfection with pIC (left) or infection with the CK/0513 virus (right). (D-E) UBL of duck (D) and ostrich (E) OASL do not change the expression of *RNase L* and several genes related to IFN signaling in DF1^OASL-/-^ cells upon infection with CK/0513 virus. (F-G) Neither human nor mouse UBL truncations induces rRNA cleavage in A549 cells upon co-transfection with pIC (F) or infection with the PR8 virus (G). (H-I) UBL of human OASL (H) and mouse Oasl2 (I) do not change the expression of *RNase L* and several genes related to IFN signaling in A549 cells. (J) UBLs of avian OASLs, but not mammalian ones, improve the binding affinity of OASL to dsRNA. Standard association and dissociation profiles of UBLs of duck OASL, ostrich OASL, human OASL and mouse Oasl2.


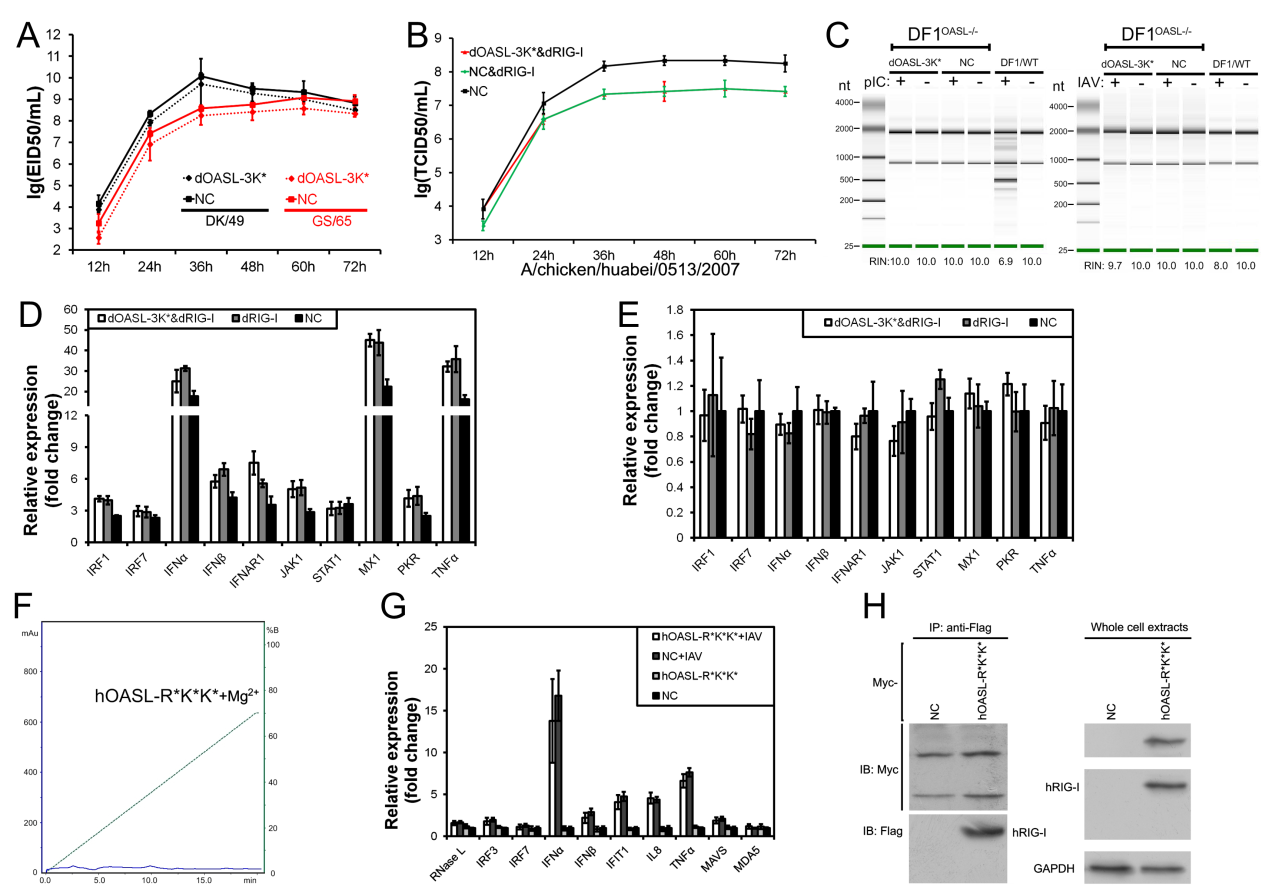


**Fig. S10 Duck and human OASL mutant (dOASL-3K*, hOASL-R*K*K*) activates and magnifies neither the OAS/RNase L nor the OASL/RIG-I pathway, and fails to inhibit the replication of CK/0513 virus in DF1 and DF1^OASL−/−^ cells.** Cells infected with virus were harvested at the indicated time points and subjected to EID50 assays or TCID50 assays on MDCK. Gene expressions were calculated relative mRNA level to that of *GAPDH* and presented as fold change against the corresponding of NC without the CK/0513 or PR8 virus infection. (A) dOASL-3K* does not affect viral replication of DK49 and GS/65 viruses in DF1 cells, which is naturally absent RIG-I. (B) Like duck and ostrich OASL, dOASL-3K* doesn’t enhance the antiviral effect of dRIG-I in DF1^OASL−/−^ cells upon CK/0513 infection. (C) Duck OASL-3K* doesn’t induce rRNA cleavage in DF1^OASL−/−^ cells. (D-E) Like duck and ostrich OASL, dOASL-3K* do not significantly change the expression of 10 genes related to the RIG-I signaling in the duck RIG-I recovery DF1^OASL−/−^ cells upon infection with CK/0513 virus. (F) The elution profiles produced by hOASL-R*K*K* protein. The 2-5A synthetase reaction was treated with alkaline phosphatase and separated using a Mono Q column. (G) hOASL-R*K*K* doesn’t significantly change the expression of *RNase L* and genes related to the RIG-I signaling in A549 cells before and after infection with PR8 virus. (H) hOASL-R*K*K* mutant doesn’t co-precipitate with hRIG-I. The data are expressed as the mean ± S.D.


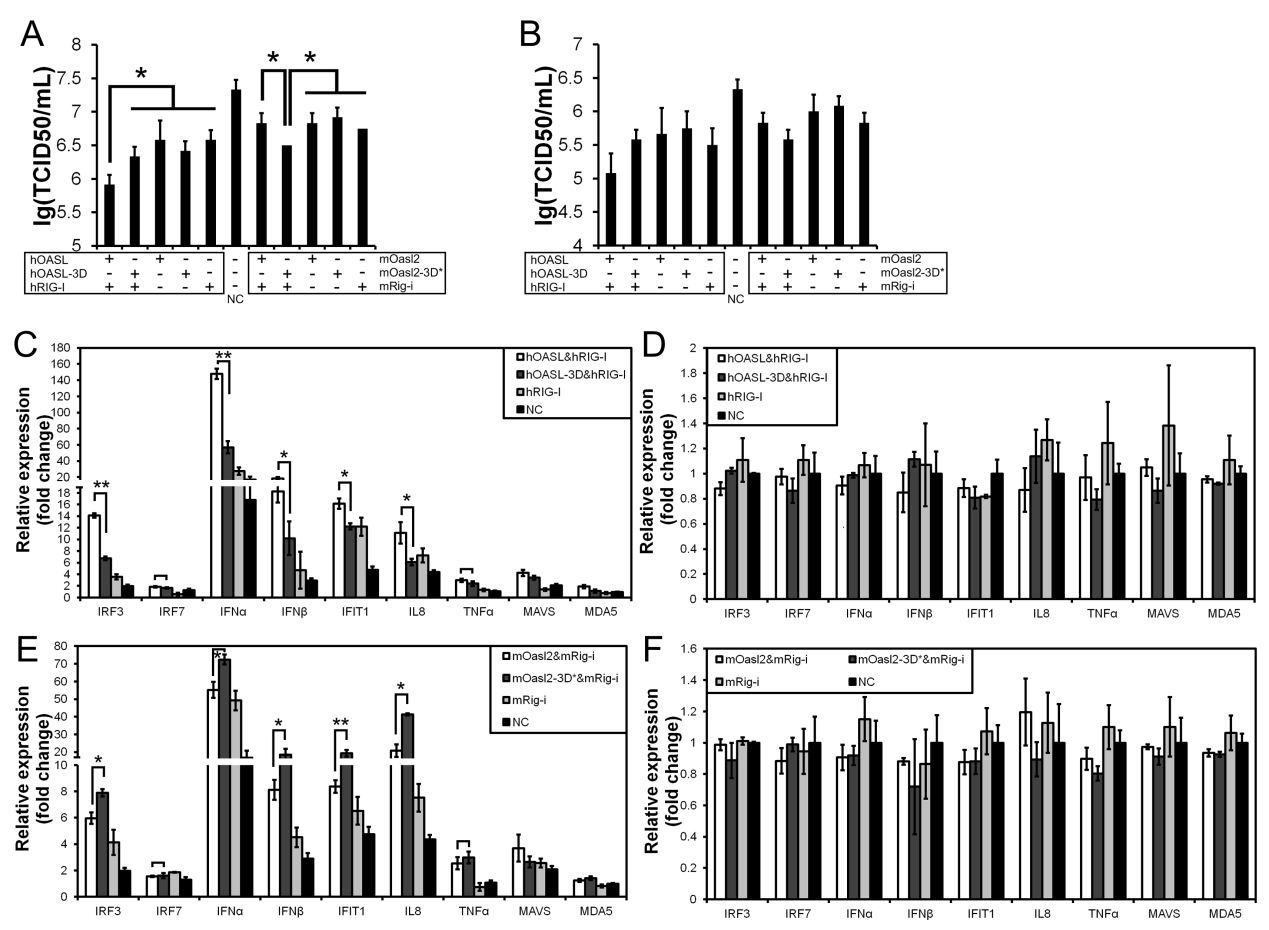


**Fig. S11 Human OASL mutant (hOASL-3D) and mouse Oasl2 (mOasl2) do not enhance the RIG-I activation and cannot magnify gene related to the RIG-I signaling like hOASL and mouse Oasl2-3D* do in A549 or HeLa cells.** Cells infected with virus were harvested at the indicated time points and subjected to TCID50 assays on MDCK cells. Gene expression in cells were calculated relative mRNA level to that of *GAPDH* and presented as fold change against the corresponding of A549 cells expressing empty vector (NC) without PR8 virus infection. The data are expressed as the mean ± S.D. **P*<0.05; ***P*<0.01. (A-B) hOASL-3D and mOasl2 present higher viral replication of PR8 virus in A549 (A) or HeLa (B) cells than hOASL and mouse Oasl2-3D* proteins when co-expressed human or mouse RIG-I, respectively. (C-F) hOASL-3D and mOasl2 co-expressed with the corresponding RIG-I proteins cannot significantly increase expression of five genes (*IRF3*, *IFNα*, *IFNβ*, *IFIT1* and *IL8*) related to the RIG-I signaling like hOASL and mOasl2-3D* do with (C, E) or without (D, F) infection of PR8 virus in A549 cells, respectively.


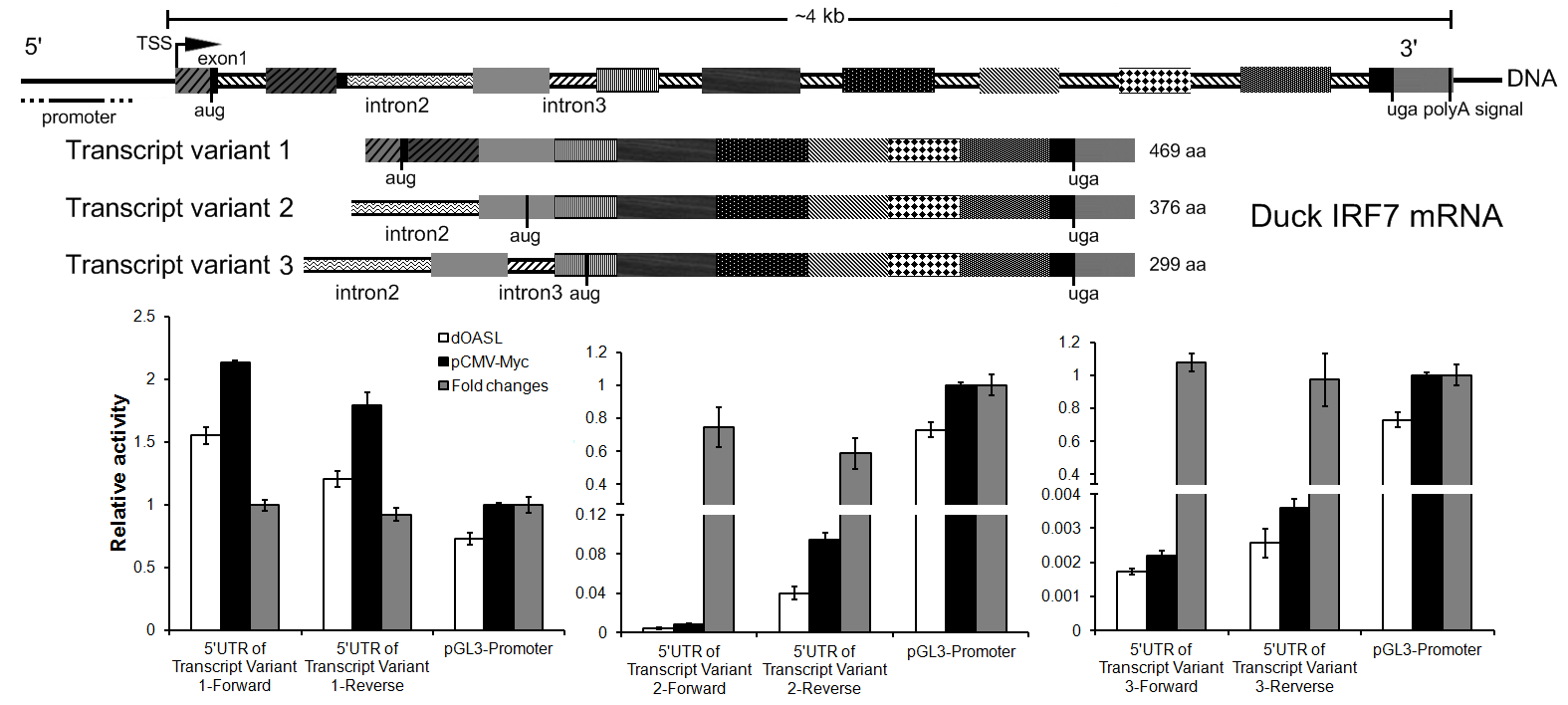


**Fig. S12 Duck OASL is not capable of binding to the 5′UTR of duck *IRF7* using luciferase assay.** Three *IRF7* transcript variants were identified from duck spleen tissue (top). Unlike mouse Oasl1, duck OASL is not capable of binding to the 5′UTR of duck *IRF7* using luciferase assay (bottom). Forward: normal sequences of duck *IRF7* 5′UTR from 5′ to 3′. Reverse: reverse complement sequences of duck *IRF7* 5′UTR.

Table S1. Primers used for quantitative PCR

Table S2. Primers used for eukaryotic plasmids construction

Table S3. Primers used for site-directed mutagenesis

Table S4. Primers used for prokaryotic plasmids construction

Table S5. Construction of the plasmids for knockout chicken OASL by CRISPR/Cas9

Table S6. Primers for genotyping OASL-KO cells

Table S7. Primers used for yeast two-hybrid analysis III

Table S1. Primers used for quantitative PCR

| Gene | Primer sequences (5′-3′) |
| --- | --- |
| cRNase L | CTACACAGAAAAGATCACAGACCTA |
|  | CCTGAATGTCTATGAAGTGACTGTA |
| cMX1 | GCACACACCCAACTGTCAGCGA |
|  | CCCATGTCCGAAACTCTCTGCGG |
| cIFNα | TACGGCATCCTGCTGCTCAC |
|  | AGAGAAGGTGGCATCCTGGG |
| cIFNβ | TGCACAGCATCCTACTGCTCTTG |
|  | GTTGGCATCCTGGTGACGAA |
| cIFNγ | AAGTCAAAGCCGCACATCAAAC |
|  | CTGGATTCTCAAGTCGTTCATCG |
| cPKR | TGGTACAGGCGTTGGTAAGAGTAAG |
|  | GGAGCACATCCGCAGGTAGA |
| cOASL | ACATCCTCGCCATCATCGA |
|  | GCGGACTGGTGATGCTGACT |
| cIFIT | CAGAATTTAATGCCGGCTATGC |
|  | TGCAAGTAAAGCCAAAAGATAAGTGT |
| cIRF1 | TCCCACCTCTGACAAAGGAC |
|  | GATGGACGTGTTCTCCACCT |
| cIRF7 | GCCTGAAGAAGTGCAAGGTC |
|  | TTTGTGCTGGAGAAGCACTG |
| cIFNAR1 | TCTTTGCCCAACAAATGTGA |
|  | CCTTCTGCCACTTTGAGGAG |
| cIFNAR2 | CTGGGTGAGTCAAAATCC |
|  | GGGTCCATATCTGAATCC |
| cJAK1 | TGCTGTCCAGACAAGAATGC |
|  | TCCTTCTCTGCCAACGTCTT |
| cSTAT1 | TAACGAGGAGCTGGTGGAGT |
|  | TTGAAAAGACTGTGCGTTCG |
| cTNFα | TGTGTATGTGCAGCAACCCGTAGT |
|  | GGCATTGCAATTTGGACAGAAGT |
| cIL6 | CGGCAGATGGTGATAAATCC |
|  | CCCTCACGGTCTTCTCCATA |
| cIL12 | ACCAGCCGACTGAGATGTTC |
|  | GTGCTCCAGGTCTTGGGATA |
| cNF-κB | TTGCTGCTGGAGTTGATGTC |
|  | TGCTATGTGAAGAGGCGTTG |
| cMAVS | CCTGACTCAAACAAGGGAAG |
|  | AATCAGAGCGATGCCAACAG |
| cMDA5 | CAGCCAGTTGCCCTCGCCTCA |
|  | AACAGCTCCCTTGCACCGTCT |
| cLGP2 | CCAGAATGAGCAGCAGGAC |
|  | AATGTTGCACTCAGGGATGT |
| Influenza A matrix | TCTCTCTATCATCCCGTCAGG |
|  | CGGTGAGCGTGAATACAAATC |
| cGAPDH | TGCCCAGAACATCATCCCA |
|  | CGGCAGGTCAGGTCAACAA |
|  | AAGTGGTCGTTGAGGGCAATG |
| FPV | ATCACAGATCCTTACCAACCGCTTA |
|  | TAGTGGTAATCATTTTTGAAGGGCT |

Table S2. Primers used for eukaryotic plasmids construction

| Gene | Primer sequences (5′-3′) | Plasmid |
| --- | --- | --- |
| dOASL/-3K*/-3D* | CGACGCGT**GCCACC**ATGGAGCTGTGGAACGTGTCCACCTCGGA | piggyBac |
|  | GGGTTTAAACTCA**CTTATCGTCGTCATCCTTGTAATC**GGAGGACGGGCAGCCGGGGTGCTGG |  |
| Δd1UBL | CGACGCGT**GCCACC**ATGGAGCTGTGGAACGTGTCCACCTCGGA | piggyBac |
|  | GGGTTTAAACTCA**CTTATCGTCGTCATCCTTGTAATC**CTCCTGGGGCTCGGTGCGCAGCAGC |  |
| dOLD | CGACGCGT**GCCACC**ATGGAGCTGTGGAACGTGTCCACCTCGGA | piggyBac |
|  | GGGTTTAAACTCA**CTTATCGTCGTCATCCTTGTAATC**GGGCTTGGCCGGCTGCACGTTCCAG |  |
| dUBL | CGACGCGT**GCCACC**ATGCGCTGCATCACCGGCGTCCAGC | piggyBac |
|  | GGGTTTAAACTCA**CTTATCGTCGTCATCCTTGTAATC**GGAGGACGGGCAGCCGGGGTGCTGG |  |
| dRIG-I | GACGCGT**GCCACC**ATGACGGCGGACGAGAAGCGGAGCC | piggyBac |
|  | GGGTTTAAACCTA**CTTATCGTCGTCATCCTTGTAATC**AAATGGTGGGTACAAGTTGGACATTTCTTC |  |
| oOASL/-3D* | CGACGCGT**GCCACC**ATGGATGGGCTGGAGACGGTGACTG | piggyBac |
|  | GGGTTTAAACTCA**CTTATCGTCGTCATCCTTGTAATC**GTGGTACATGGGTACAGCGGTGTAG |  |
| Δo1UBL | CGACGCGT**GCCACC**ATGGATGGGCTGGAGACGGTGACTG | piggyBac |
|  | GGGTTTAAACTCA**CTTATCGTCGTCATCCTTGTAATC**CTCCTGGGTCTCGGTGTGCAGCAGC |  |
| oOLD | CGACGCGT**GCCACC**ATGGATGGGCTGGAGACGGTGACTG | piggyBac |
|  | GGGTTTAAACTCA**CTTATCGTCGTCATCCTTGTAATC**GGGCTGGGCTGGCTGCACATCCCAG |  |
| oUBL | CGACGCGT**GCCACC**ATGCAGCCCGTGACAGTGGAGATAAAGC | piggyBac |
|  | GGGTTTAAACTCA**CTTATCGTCGTCATCCTTGTAATC**GTGGTACATGGGTACAGCGGTGTAG |  |
| dOASL/-3K*/-3D* | CCTCGAGGTATGGAGCTGTGGAACGTGTCCACCT | pCMV-Myc |
|  | TTGCGGCCGCTCAGGAGGACGGGCAGCCGGGGTGCTGG |  |
| Δd1UBL | CCTCGAGGTATGGAGCTGTGGAACGTGTCCACCT | pCMV-Myc |
|  | TTGCGGCCGCTCACTCCTGGGGCTCGGTGCGCAGCAGC |  |
| dOLD | CCTCGAGGTATGGAGCTGTGGAACGTGTCCACCT | pCMV-Myc |
|  | TTGCGGCCGCTCAGGGCTTGGCCGGCTGCACGTTCCAG |  |
| dUBL | CCTCGAGGTATGCGCTGCATCACCGGCGTCCAGC | pCMV-Myc |
|  | TTGCGGCCGCTCAGGAGGACGGGCAGCCGGGGTGCTGG |  |
| oOASL/-3D* | CCGTCGACCATGGATGGGCTGGAGACGGTGACTG | pCMV-Myc |
|  | TTGCGGCCGCTCAGTGGTACATGGGTACAGCGGTGTAG |  |
| Δo1UBL | CCGTCGACCATGGATGGGCTGGAGACGGTGACTG | pCMV-Myc |
|  | TTGCGGCCGCTCACTCCTGGGTCTCGGTGTGCAGCAGC |  |
| oOLD | CCGTCGACCATGGATGGGCTGGAGACGGTGACTG | pCMV-Myc |
|  | TTGCGGCCGCTCAGGGCTGGGCTGGCTGCACATCCCAG |  |
| oUBL | CCGTCGACCCAGCCCGTGACAGTGGAGATAAAGC | pCMV-Myc |
|  | TTGCGGCCGCTCAGTGGTACATGGGTACAGCGGTGTAG |  |
|  | TTGCGGCCGCCTAGTCGATTACCCTAATGAGTATGATG |  |

Table S3. Primers used for site-directed mutagenesis

| Gene | Primer sequences (5′-3′) |
| --- | --- |
| dOASL-3K* | F:GCTTCACTGAGCTGCAG**G**AGAAGTTCGTG**G**AGCGCTGCCCCGCC**G**AGCTGAA |
|  | R:GGCGCAGCAGGTTCTTCAGCT**C**GGCGGGGCAGCGCT**C**CACGAACTTCT**C**CTGCA |
| dOASL-3D* | F1:CACGGCTCTGCGCAACAACTCGG**C**CGCCG**C**CGTGGTGAT |
|  | R1:GGAGAAGCAGCTGAGGAAGATCACCACG**G**CGGCG**G**CCGAGTT |
|  | F2:CTCTCCTCCAACGGCGAGTCCATCGAAGTCG**C**CGTCCT |
|  | R2:GCATCACCTGGCCCAGGGCATCGTAGGTGGGCAGGACG**G**CGACTTCGAT |
| oOASL-3D* | F1:GGCGGCTCAGCAGGCAAGGGCACGGCCCTGCAGAACAACTCTG**C**CGCTG**C**CGTGG |
|  | R1:CTGATCCTGGTAGCTGGAGAAGCAGCTGAGGAAGAGCACCACG**G**CAGCG**G**CAGAG |
|  | F2:CTTAGCCTTACTCTCCAGTCCAAGTCATGTGCGGAGTCCATCGATGTAG**C**TGTCC |
|  | R2:CGCAGCGTCCTGAGTCACCTGGCCCAAGGCATCATAGGCAGGCAGGACA**G**CTACA |

Table S4. Primers used for prokaryotic plasmids construction

| Gene | Primer sequences (5′-3′) | Molecular weight (k Da) |
| --- | --- | --- |
| dOASL/-3K*/-3D* | CCCATATGATGGAGCTGTGGAACGTGTCCACCT | 58 |
|  | CCTCGAGGGAGGACGGGCAGCCGGGGTGCTGG |  |
| Δd1UBL | CCCATATGATGGAGCTGTGGAACGTGTCCACCT | 48 |
|  | CCTCGAGTTACTCCTGGGGCTCGGTGCGCAGCAGCA |  |
| dOLD | CCCATATGATGGAGCTGTGGAACGTGTCCACCT | 38 |
|  | CCTCGAGTTAGGGCTTGGCCGGCTGCACGTTCCAG |  |
| dUBL | CCCATATGCCCGTGACGCTGGAGGTGAGGGGGC | 20 |
|  | CCTCGAGGGAGGACGGGCAGCCGGGGTGCTGG |  |
| oOASL/-3D* | CCCATATGATGGATGGGCTGGAGACGGTGACTG | 60 |
|  | CCTCGAGGTGGTACATGGGTACAGCGGTGTAG |  |
| Δo1UBL | CCCATATGATGGATGGGCTGGAGACGGTGACTG | 48 |
|  | CCTCGAGCTCCTGGGTCTCGGTGTGCAGCAGC |  |
| oOLD | CCCATATGATGGATGGGCTGGAGACGGTGACTG | 38 |
|  | CCTCGAGGGGCTGGGCTGGCTGCACATCCCAG |  |
| oUBL | CCCATATGCAGCCCGTGACAGTGGAGATAAAGC | 22 |
|  | CCTCGAGGTGGTACATGGGTACAGCGGTGTAG |  |
| hUBL | CCCATATGGACATCCACTTGACAGTGGAGCAGA | 19 |
|  | CCAAGCTTACTGGCTGGAAACAGAGCCTCTCCT |  |
| mUbl | CCCATATGGATGTTCAGGTGAGAGTGAAACAAA | 18 |
|  | CCTCGAGGTCGATTACCCTAATGAGTATGATG |  |
| dOLD-CFK1* | CCCATATGATGGAGCTGTGGAACGTGTCCACCT | 38 |
|  | CCTCGAGGGGCTTGGCCGGCTGCACGTTCCAGGGCTGGACGCCGGT**TT**TG**A**AGC**A**CAT |  |
| dOLD-CFK2* | CCCATATGATGGAGCTGTGGAACGTGTCCACCT | 38 |
|  | CCTCGAGGGGCTTGGCCGGCTGCACGTTCCAGGGCTGGACGCC**TT**TGA**A**GCAGCGCAT |  |

Table S5. Construction of the plasmids for knockout chicken OASL by CRISPR/Cas9

| Gene | Primer sequences (5′-3′) | Targeting region |
| --- | --- | --- |
| Chicken OASL | CACCG***TACCCGGGGGAAATAGCGGG*** | Upstreams |
|  | AAAC***CCCGCTATTTCCCCCGGGTA***C |  |
| Chicken OASL | CACCG***CCGGCGATGACCGCAACCCG*** | Downstreams |
|  | AAAC***CGGGTTGCGGTCATCGCCGG***C |  |

Table S6. Primers for genotyping OASL-KO cells

| Detecting region | Primer sequences (5′-3′) |
| --- | --- |
| Chicken OASL upstreams | TCCATGTTTCATTTCCATTCACGTT |
|  | CTGGAGAAGCAGTTGATGAAGAGCA |
| Chicken OASL downstreams | TGGGAATAAAAGCTGTGAACTCTGG |
|  | CCTCGGCTACACTTTCGTTTCCT |
| Inside of chicken OASL | AGAAGAACTTTGTGAAGTGGCGCCC |
|  | CCACCAAGTCCCAGTTCTTGCCT |
| Inside of chicken OASL | TCCTTCGGAGTCAGCATCACCAGTC |
|  | GGGGGATGCACCACTCCTTCTCTAT |
| Chicken OASL knockout | TCCATGTTTCATTTCCATTCACGTT |
|  | AGCGGTGTGTGCCCACCCACGA |

Table S7. Primers used for yeast two-hybrid analysis

| Gene | Primer sequences (5′-3′) | Plasmid |
| --- | --- | --- |
| dOASL/-3K*/-3D* | CCCATATGATGGAGCTGTGGAACGTGTCCACCT | pGADT7 |
|  | CCTCGAGGGAGGACGGGCAGCCGGGGTGCTGG |  |
| Δd1UBL | CCCATATGATGGAGCTGTGGAACGTGTCCACCT | pGADT7 |
|  | CCTCGAGTTACTCCTGGGGCTCGGTGCGCAGCAGCA |  |
| dOLD | CCCATATGATGGAGCTGTGGAACGTGTCCACCT | pGADT7 |
|  | CCTCGAGTTAGGGCTTGGCCGGCTGCACGTTCCAG |  |
| dUBL | CCCATATGCCCGTGACGCTGGAGGTGAGGGGGC | pGADT7 |
|  | CCTCGAGGGAGGACGGGCAGCCGGGGTGCTGG |  |
| dRIG-I | CCCATGGAGATGACGGCGGACGAGAAGCGGAGCC | pGBKT7^†^ |
|  | CGAGCTCCTAAAATGGTGGGTACAAGTTGGACATT |  |
| dRIG-I-CARD | CCCATGGAGATGACGGCGGACGAGAAGCGGAGCC | pGBKT7^†^ |
|  | CGAGCTCCTATCTTATATCCCACAGTTCACTTGCA |  |
| hRIG-I | CCCATATGATGACCACCGAGCAGCGACGCAGCC | pGBKT7 |
|  | GCGTCGACTCATTTGGACATTTCTGCTGGATCAAAT |  |

^†^ Mutant vector that bring in *Sac* I restriction site derived from the previous pGBKT7 plasmid
